# Robust fractional-order adaptive cascaded strategy for mitigating load frequency deviations in renewable thermal hybrid systems

**DOI:** 10.1038/s41598-025-21454-z

**Published:** 2025-10-09

**Authors:** Youssef M. Abdel Halim, Othman A. M. Omar, Mahmoud A. Attia, Ibrahim Mohamed Diaaeldin, Ahmed O. Badr, Mohamed A. Afifi

**Affiliations:** 1 Electrical Power and Machines Department, Faculty of Engineering, Ain Shams University, Cairo, 11517 Egypt; 2 Engineering Physics and Mathematics Department, Faculty of Engineering, Ain Shams University, Cairo, 11517 Egypt

**Keywords:** Fractional order derivatives, Adaptive control, Hybrid power systems, Frequency control, Energy science and technology, Engineering, Mathematics and computing

## Abstract

In modern power systems, the increasing share of renewable energy sources (RES) is primarily due to their environmental benefits and sustainability. However, because RES like wind and solar are inherently variable and intermittent besides interfacing with the grid mainly through power electronic devices, they act mainly as power injectors rather than conventional synchronous generators. This results in a reduction of system inertia, intensifying frequency stability challenges. To address this problem, this paper proposes a robust cascaded control strategy for mitigating load frequency deviations in a two-area hybrid renewable–thermal power system. The proposed controller integrates a fractional-order adaptive PID (FOAPID) with a harmony search-optimized PIDA (HS-PIDA), combining the adaptability of Caputo fractional derivatives and model reference adaptive control with the accelerated derivative action of PIDA to enhance dynamic performance. The system model incorporates solar PV, wind, and conventional thermal units, capturing nonlinearities and renewable variability. Six case studies are performed to assess robustness against step and dynamic load changes, renewable intermittency, and parameter uncertainties. Results reveal that the proposed controller reduces settling time for frequency deviations to as low as 3.1 s compared to 14–18 s for TLBO PIDA, SCA PIDA, and Fuzzy PID. Undershoot values are decreased by more than 80% across all cases, while steady-state errors are eliminated where competing controllers failed to converge. Error indices confirm superiority, with SSE reductions of up to 99% and RMSE reductions of up to 97% compared to alternative controllers. Against Marine Predator Algorithm (MPA)-Cascaded PIDA, the proposed scheme further improves overshoot and undershoot by up to 89.85% and achieves faster convergence, proving its effectiveness under diverse operating conditions.

## Introduction

The integration of renewable energy sources (RES) into modern power systems is increasing day by day^1,2^ due to their various merits such as having no carbon dioxide emissions, good impact on environment, economical operation cost, also they are clean resources, and highly available^3,4^. A hybrid power system combines various power generation sources, including renewables like solar and wind, alongside conventional sources such as diesel generators, to deliver a stable and sustainable electricity supply^5^. The growing share of renewables in hybrid power systems poses challenges for maintaining frequency stability due to their stochastic nature and their reliance on power electronic devices to interface with the grid, which results in reduced system inertia^6^. Therefore, robust load frequency control (LFC) is needed to maintain frequency stability in hybrid power systems by minimizing the frequency deviations and tie-line power under varying system operating conditions.

The operation of any LFC in a multi-area electrical network mainly depends on the controller’s action with respect to different sub systems presented in LFC model. When a load disturbance occurs, the governor’s reference power setting is adjusted by using the area control error (ACE) as the controller’s input to stabilize system frequency and tie-line power flow fluctuations^7–9^. Large oscillations in system frequency and tie line power flow in interconnected power systems can cause network instability and reduce the life of both power generation equipment and home appliances, so to solve this problem various controllers have been studied in the literature to improve the efficiency of load frequency control. A bat inspired algorithm (BIA) based MPC was implemented in^10^ to improve the damping of oscillations in two-area hydro-thermal power system. The BIA based MPC was compared to the conventional PI controller and the genetic algorithms (GA) based PI controller and outperformed both. In^11^, the authors implemented a recurrent artificial neural network (ANN) based LFC in a two-area power system connected through high voltage direct current (HVDC) tie-line. The performance of recurrent ANN-based LFC was found to be better than conventional proportional-integral (PI) controllers in terms of dynamic response indices such as oscillations and settling time. In^12^, the authors address simultaneous voltage and frequency control for multisource power systems that include stochastic wind sources. They propose a new MPC design optimized using the harris hawks optimization (HHO) algorithm and examine its effectiveness in ensuring system stability and performance in the face of wind power fluctuations.

Although the mentioned controllers exhibited a suitable system response, the classical controllers such as PI and proportional-integral-derivative (PID (are widely utilized in LFC due to their simplicity and efficiency^13^. Among different LFC strategies, the PI controller remains the most commonly utilized^14^. It is also worth mentioning that various optimization algorithms were presented to adjust PI and PID parameters for controllers used in LFC problems such as particle swarm optimization^15^, genetic algorithm^16^, harmony search algorithm^17^, and catch fish algorithm^18^.

While conventional control strategies have been widely used for load frequency control, their performance can be limited under system variations and uncertainties, which motivates the adoption of adaptive control techniques capable of adjusting to changing operating conditions. In^19^, an adaptive model predictive controller (AMPC) was developed to improve the LFC in a two area power system including thermal generation, RES such as PV and wind generation, fuel cell, and battery storage while considering physical constraints such as the reheat turbine (RT), the time delay (TD), the generation rate constraint (GRC), and the governor dead band (GDB). The results proved the superiority and robustness of the AMPC compared to the conventional model predictive control (MPC). The authors of^20^ developed an indirect adaptive fuzzy logic control (IAFLC) for a real three area power system located in the Arabian Gulf region.

Because conventional PID controllers may lack the flexibility to handle highly variable and uncertain system dynamics, fractional-order PID (FOPID) controllers, which are derived from PID controllers, have been developed. Due to FOPID controllers several advantages such as excellent set point tracking a disturbance handling ability numerous researchers proposed them to address LFC issues in multi-area power systems^21,22^. The authors of^23^ have developed an interval fractional-order PID using stability boundary locus method for a two-area power system to enhance the system peak to peak oscillations and settling time while considering system parametric uncertainty using Kharitonov theorem. Another recent controller studied by many researchers and derived from the conventional PID controller is proportional-integral-derivative-accelerated (PIDA) controller which is utilized in high order systems in order to generate smooth overshoots and faster settling times^24^. The authors of^25,26^ used different optimization techniques to optimize PIDA controller parameters which are teaching learning-based optimization (TLBO), sine–cosine algorithm (SCA), and harmony search (HS). It was found that TLBO-PIDA controllers have better frequency stability compared to SCA-PIDA and HS-PIDA controllers. Paper^27^ presents a fractional-order-fuzzy-PID (FOFPID) controller optimized by modified harris hawks optimizer (mHHO) for LFC in a multi-micro-grid (MMG) including renewable energies.

It is noteworthy that one of the recent approaches employed for LFC in multi area systems is cascaded control because it has better disturbance elimination compared to single loop control. In^28^, the authors implemented a cascaded PIDA controller optimized by the marine predator algorithm (MPA). The results demonstrated that the cascaded PIDA controller outperforms the single-structure MPA-PIDA controller in minimizing both the maximum and minimum overshoots in frequency and tie-line power flow deviations. MPA was used again in^29^ to optimize a novel P-P-FOPID cascaded controller for LFC. Furthermore, in^30^ a proportional-derivative (PD) controller in cascading with fractional order PID controller was proposed for deregulated two area power system and it was tested against fractional order PID controller, then it was found that the cascading strategy has a better transient response under 1% load disturbance. The authors of^31^ developed a cascaded tilt derivative filter-tilt integral derivative filter (TDF-TIDF) controller optimized using the dragonfly algorithm (DFA) for LFC in an isolated hybrid microgrid with renewable and conventional sources. Compared to PIDF, TIDF, and PDF-PIDF controllers, the DFA-based TDF–TIDF demonstrated superior capability in rapidly damping oscillations with system nonlinearities. Moreover, a novel PI-(1 + FOPID) cascade controller fine-tuned using wild horse optimizer (WHO) was presented in^32^ for LFC in four power systems: a two-area non-reheated thermal system with and without GDB, a two-area multi-source system, and a three-area hydro-thermal system with GRC. The WHO-based PI-(1 + FOPID) was found to have superior performance over recent methods by reducing overshoot, undershoot, and settling time in frequency deviations and tie-line power oscillations. Another study^33^ used WHO to optimize a novel control structure termed the fractional-order proportional derivative—(one + fractional order integrator) (FOPD-(1 + FOI)) cascade controller to enhance automatic generation control (AGC) for the linked power system load frequency management (LFM).

Previous studies have proposed various strategies to address the LFC problem, including adaptive controllers, fractional-order controllers, PIDA controllers, and cascaded controllers. At the same time, the increasing penetration of RES introduces significant frequency stability challenges, creating a growing need for robust and reliable load frequency controllers. Motivated by this, the authors combined these strategies to leverage their advantages and developed a new adaptive fractional order PID controller cascaded with a harmony search optimized PIDA controller for LFC in a two-area power system incorporating RES, considering different disturbances of system dynamics.

The main contribution lies in developing a novel cascaded FOAPID/HS-PIDA control structure that leverages Caputo fractional derivatives and adaptive model reference control for improved precision, combined with accelerated derivative action for enhanced transient performance. The paper introduces rigorous mathematical modeling of the controller in both time and Laplace domains, alongside a comprehensive hybrid system model capturing load, tie-line, and renewable dynamics. Six case studies systematically evaluate the proposed design against conventional and advanced controllers, covering disturbances such as step load changes, dynamic variations, PV/wind fluctuations, and parameter uncertainty. A statistical analysis across cases validates the controller’s superior robustness, precision, and adaptability, making it highly suitable for modern renewable-integrated power grids.

The manuscript is organized as: “Introduction” section provides the justified introduction and the literature review analysis, “Mathematical preliminaries of cascaded control strategy” section illustrates the mathematical preliminaries of our study, “Hybrid power system under study” section shows the modeling part for the established hybrid power system understudy, “Results and discussions” section displays the results obtained with detailed required discussions, and finally conclusions are described at “Conclusions” section.

## Mathematical preliminaries of cascaded control strategy

Fractional differential equations have garnered a lot of attention recently because the fractional-order system response eventually converges to the integer-order system response. The advantages of fractional derivatives include a higher degree of model flexibility and a useful tool for describing the characteristics of many real-world processes and dynamical systems.

Definition 2.1.1 Suppose that ϕ, t > 0, ϕ, t ∈ R. The fractional operator (${\text{D}}_{{\text{t}}}^{\upphi }$), defined as^34^:

1 $${\text{D}}_{{\text{t}}}^{\upphi } {\text{f}}\left( {\text{t}} \right) = \left\{ {\begin{array}{*{20}l} {\frac{1}{{\Gamma \left( {{\text{n}} -\upphi } \right)}}\left[ {\mathop \smallint \limits_{0}^{{\text{t}}} \left( {{\text{t}} -\upzeta } \right)^{{{\text{n}} -\upphi - 1}} {\text{f}}^{{\left( {\text{n}} \right)}} \left(\upzeta \right){\text{d}}\upzeta } \right],} \hfill & {{\text{where}}\;0 \le {\text{n}} - 1 <\upphi < {\text{n}} \in {\text{N}}_{{\text{R}}} } \hfill \\ {\frac{{{\text{d}}^{{\text{n}}} }}{{{\text{dt}}^{{\text{n}}} }}{\text{f}}\left( {\text{t}} \right),} \hfill & {\upphi = {\text{n}} \in {\text{N}}_{{\text{R}}} } \hfill \\ \end{array} } \right.$$

This operator is called the Caputo fractional derivative or Caputo fractional differential operator of order ϕ and N_R_ is the set of positive integer numbers. Lemma 2.1.1 Let us assume that the following nonlinear dynamical system driven by Caputo fractional derivative:

2 $${\text{D}}_{{\text{t}}}^{\upphi } {\text{x}}\left( {\text{t}} \right) = {\text{f}}\left( {{\text{x}},{\text{u}}} \right);{\text{y}}\left( {\text{t}} \right) =\uplambda \left( {{\text{x}},{\text{u}}} \right){\text{ for t}} > 0,{\text{x}}^{{\left( {\text{k}} \right)}} \left( {{\text{t}}_{0} } \right) = {\text{x}}_{{{\text{k}}0}} \;{\text{and}}\;{\text{k}} = 0,1, \ldots ,{\text{n}} - 1$$

where x ∈ ${\mathbb{R}}^{{\text{n}}}$ is the state vector, f: ${\mathbb{R}}^{{\text{n}}} \times {\mathbb{R}}^{{\text{m}}} \to {\mathbb{R}}^{{\text{n}}} { }$ is a Lipschitz continuous function, $x_{k0}$ ∈ ${\mathbb{R}}^{{\text{n}}}$ are the initial conditions at ${\text{t}}_{0} = 0$ , $y$ ∈ ${\mathbb{R}}^{{\text{p}}}$ donates the available output from the system, λ: ${\mathbb{R}}^{{\text{n}}} \times {\mathbb{R}}^{{\text{m}}} \to {\mathbb{R}}^{{\text{n}}}$ is a continuous function of t, and $u$ ∈ ${\mathbb{R}}^{{\text{m}}}$ is the vector input, ϕ $= \left[ {\phi_{1} ,\phi_{2} , \ldots ,\phi_{n} } \right]^{T}$, $0 < \phi_{1} ,\phi_{2} , \ldots ,\phi_{n} < n$. As f and λ are Lipschitz continuous functions, then system (2) has a unique solution on ${\mathbb{R}}^{{\text{n}}}$.

Laplace transformation is applied to get a solution for dynamic system (2) as:

3 $${\text{s}}^{\upphi } {\text{X}}\left( {\text{s}} \right) - \mathop \sum \limits_{{{\text{k}} = 0}}^{{{\text{n}} - 1}} {\text{s}}^{{\upphi - {\text{k}} - 1}} {\text{x}}^{{\left( {\text{k}} \right)}} \left( 0 \right) = {\text{F}}\left( {\text{s}} \right),$$

4 $${\text{X}}\left( {\text{s}} \right) = \frac{1}{{{\text{s}}^{\upphi } }}{\text{F}}\left( {\text{s}} \right) + \mathop \sum \limits_{k = 0}^{n - 1} s^{{ - \left( {k + 1} \right)}} x_{k0} .$$

Taking inverse Laplace transform:

5 $${\text{x}}\left( {\text{t}} \right) = \frac{{{\text{t}}^{n - 1} }}{{\left( {n - 1} \right)!}}x_{{\left( {n - 1} \right)0}} + \cdots + x_{00} + \mathop \smallint \limits_{0}^{{\text{t}}} \frac{{\uptau ^{{\upphi - 1}} }}{{\Gamma \left(\upphi \right)}}{\text{f}}\left( {{\text{t}} -\uptau } \right){\text{d}}\uptau .$$

where Г is gamma function.

### Mathematical preliminaries of PIDA controller

One of the latest controllers for electrical power system control that has been thoroughly examined is the PIDA controller. The generalized transfer function of n^th^ order type for this controller can be expressed as^35^:

6 $${\text{y}}\left( {\text{s}} \right) = \frac{{{\text{a}}_{0} {\text{s}}^{{\text{n}}} + {\text{a}}_{1} {\text{s}}^{{{\text{n}} - 1}} + \cdots + {\text{a}}_{{\text{n}}} { }}}{{{\text{b}}_{0} {\text{s}}^{{\text{n}}} + {\text{b}}_{1} {\text{s}}^{{{\text{n}} - 1}} + \cdots + {\text{b}}_{{\text{n}}} }}{\text{x}}\left( {\text{s}} \right),$$

where $\left( {{\text{a}}_{0} ,{\text{a}}_{1} , \ldots ,{\text{a}}_{{\text{n}}} ,{\text{b}}_{0} ,{\text{b}}_{1} , \ldots ,{\text{b}}_{{\text{n}}} } \right)$ are the controller designing parameters.

Numerous research employing various metaheuristic optimization methodologies focus on choosing the controllers’ parameters for the best possible system control. This kind of controller can be approximate of as a sum of parallel fractional order PID controllers in the typical scenario as:

7 $$\frac{{{\text{a}}_{0} {\text{s}}^{{\text{n}}} + {\text{a}}_{1} {\text{s}}^{{{\text{n}} - 1}} + \cdots + {\text{a}}_{{\text{n}}} }}{{{\text{b}}_{0} {\text{s}}^{{\text{n}}} + {\text{b}}_{1} {\text{s}}^{{{\text{n}} - 1}} + \cdots + {\text{b}}_{{\text{n}}} }} \cong \mathop \sum \limits_{{{\text{i}} = 1}}^{{\text{n}}} {\text{k}}_{{{\text{p}}_{{\text{i}}} }} + {\text{k}}_{{{\text{i}}_{{\text{i}}} }} {\text{s}}^{{ -\uplambda _{{\text{i}}} }} + {\text{k}}_{{{\text{d}}_{{\text{i}}} }} {\text{s}}^{{\upmu _{{\text{i}}} }} .$$

### Mathematical preliminaries of FOAPID controller

**Fig. 1:**
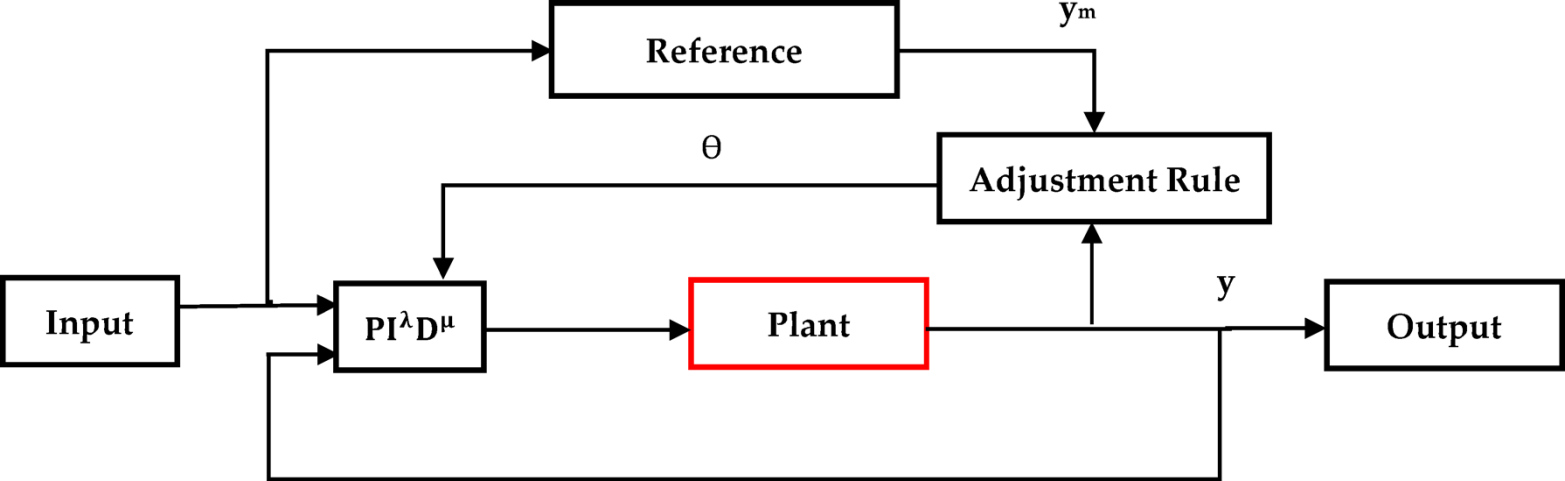
Basic principles of FOAPID control system.

This section examines the use of fractional calculus to model reference adaptive control (APID) to generate fractional-order model reference adaptive (FOAPID) controller. This is made though using adaptive control in the model reference with the fractional-order PID controller, as illustrated in Fig. 1. The model reference adaptive system is one of the main techniques for adaptive control. In this approach, the desired performance is expressed in terms of the reference model, which describes the desired input–output characteristics of the closed-loop system^36^. The controller’s parameters are then adjusted based on the difference between the reference model’s output and the system output. Two loops are used in Fig. 1 to demonstrate these basic concepts: an outer loop that adjusts the parameters in the inner loop which provides the normal control feedback.

The parameters are assumed to change more slowly than the system’s other variables when reference adaptive control is modeled using the gradient approach. The estimation of the sensitivity derivatives required for the adaptation depends on this assumption, which admits quasi stationary treatment.

Let ($e$) represent the difference error between the reference output ($y_{m}$) and the system output ($y$). Let ($\theta$) represent the parameters that need to be adjusted. Using the objective function for minimization ($J$):

8 $${\text{J}}\left(\uptheta \right) = \frac{1}{2}{\text{e}}^{2} ,\quad {\text{e}} = {\text{y}} - {\text{y}}_{{\text{m}}} .$$

and the rule for adjusting the parameters to change them in the direction of J’s negative gradient is that

9 $$\frac{{{\text{d}}\uptheta }}{{{\text{dt}}}} = -\uplambda \frac{{\partial {\text{J}}}}{{\partial\uptheta }} = -\uplambda {\text{e}}\frac{{\partial {\text{e}}}}{{\partial\uptheta }}.$$

The derivative $\partial e/\partial \theta$, or the system’s sensitivity derivative, can be assessed assuming that $\theta$ is constant if it is assumed that the parameters change substantially more slowly than the other variables.

### The proposed cascaded controller scheme

**Fig. 2:**
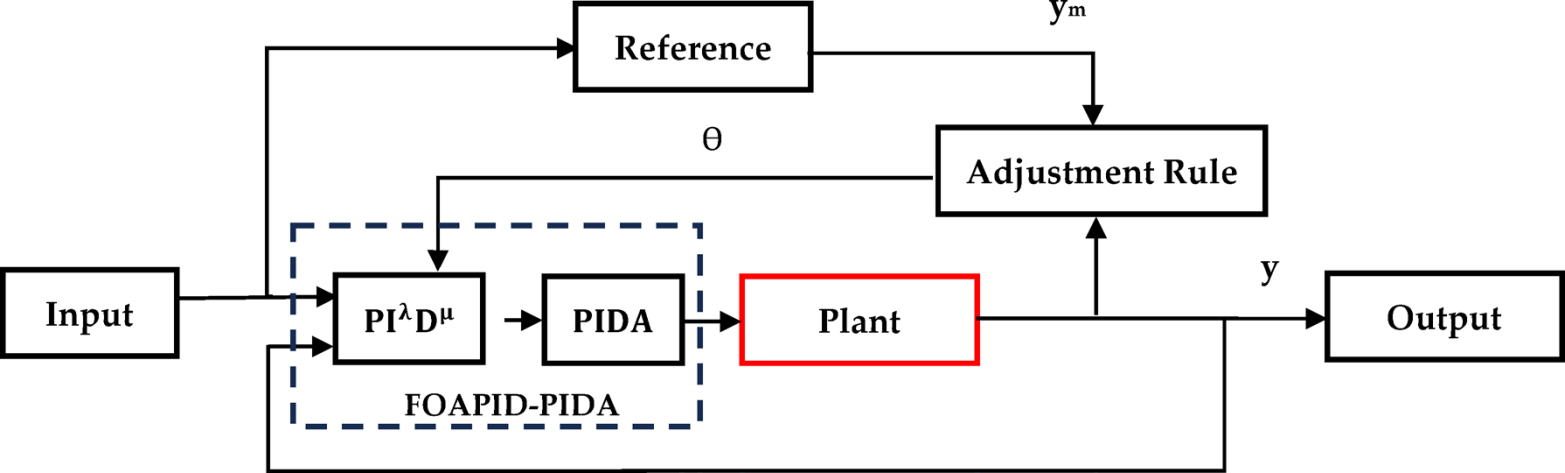
The proposed FOAPID-PIDA controller block diagram.

The proposed novel controller, abbreviated by FOAPID-PIDA, is constructed through utilizing the previous defined controllers: the FOAPID and the PIDA in cascaded order as presented in Fig. 2. While this interconnection will increase the complexity of the system dynamics, it will combine the advantages and disadvantages of both controllers resulting in giving stronger control strategy with fast and smooth response of controlled outputs for different applications. In this manuscript, the FOAPID-PIDA is tested through applying to a hybrid double area power system to control both areas’ frequency deviations under classical disturbances and RES natural variations which require strong control to counteract under these sudden disturbances.

## Hybrid power system under study

**Fig. 3:**
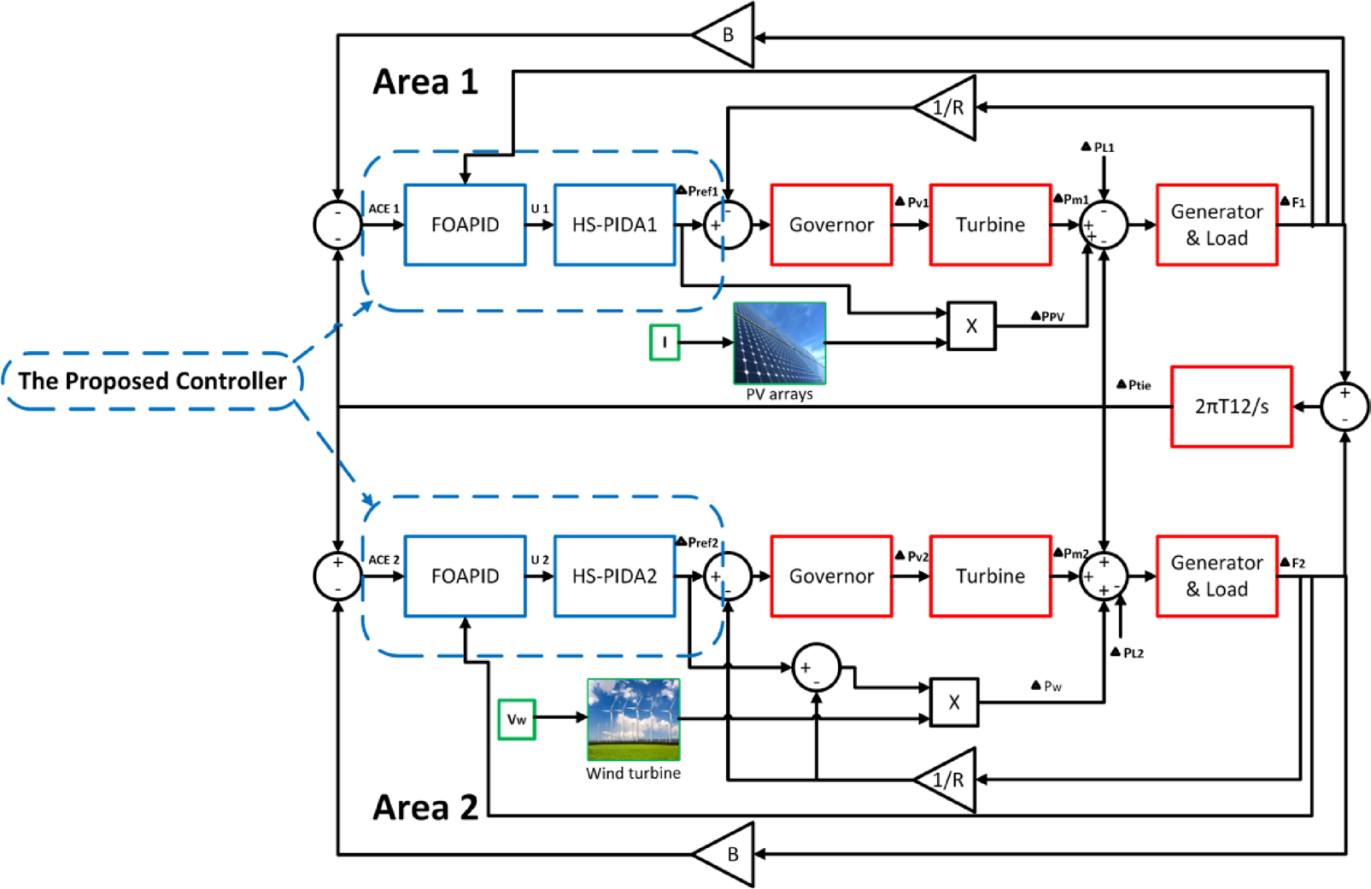
Block diagram of proposed two-area hybrid power system.

**Fig. 4:**
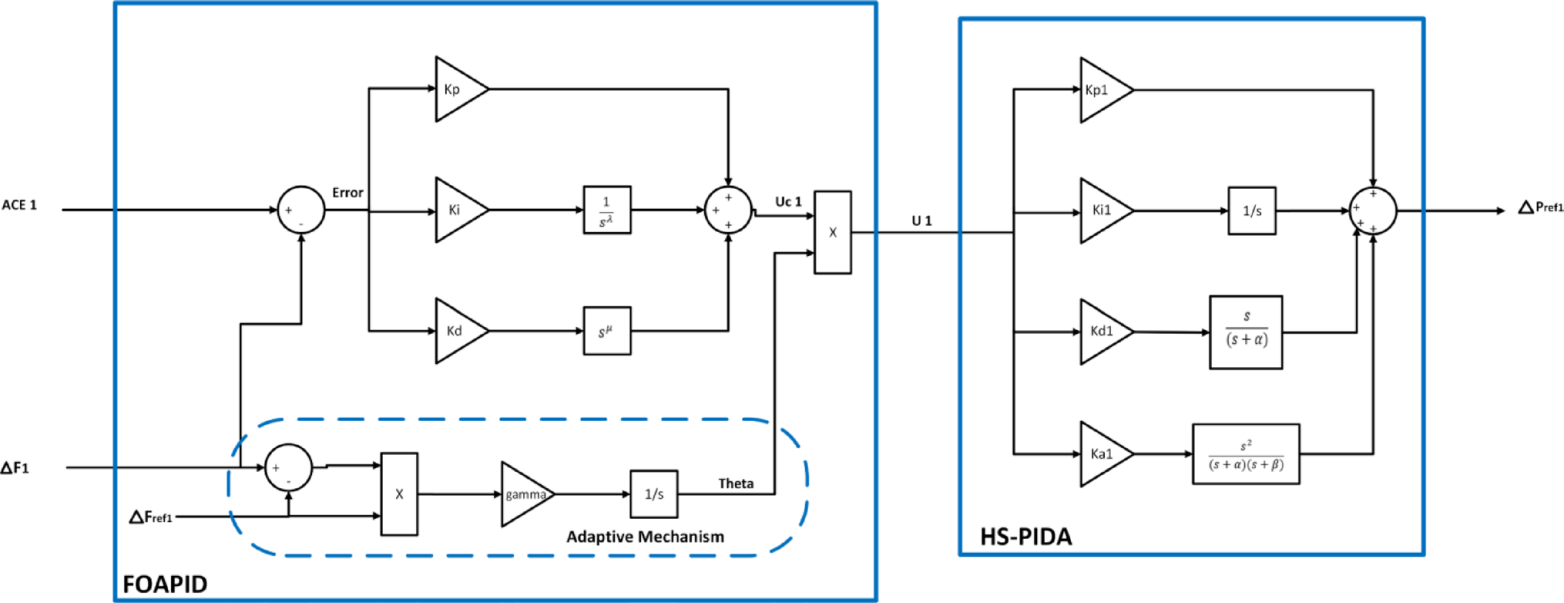
Block diagram of FOAPID/HS-PIDA controller on Laplace domain*.*

A control area is a group of coherent generators with LFC. Each region is linked to adjacent areas via tie lines. The primary purpose behind the LFC is to keep a constant frequency against any load disturbance. In this study, a dual-area hybrid power system is modelled as shown in Fig. 3 and the system parameters are summarized in Table 1^37,38^. The system governor, the non-reheat steam turbine, and power system (generator and load) models used in the simulations were linearized by using first order transfer functions. In addition, the proposed controller PIDA part gains are set using harmony search algorithm (HS) as indicated at^25,26^. The whole controller scheme block diagram is depicted in Fig. 4. Furthermore, the power system simulation used in this study was performed using MATLAB R2021a/Simulink.

**Table 1:** System parameters.

Parameter	Value	Parameter	Value
$k_{p}$	20	$a_{10}$	126.8
$k_{i}$	0.06	$a_{11}$	875.6
$k_{d}$	0.95	$a_{12}$	608.5
$\lambda$	0.5	$a_{13}$	200
$\mu$	0.5	$a_{20}$	141.3
$\gamma$	200	$a_{21}$	499.8
$k_{l}$	46	$a_{22}$	574.4
$k_{w1}$	1	$a_{23}$	500
$k_{w2}$	0.34	$b_{10}$	1
$k_{w3}$	0.4	$b_{11}$	657.3
$T_{g}$	0.08 s	$b_{12}$	1338.5
$T_{t}$	0.3 s	$b_{13}$	0.01
$T_{l}$	20 s	$b_{20}$	1
$T_{12}$	0.5 s	$b_{21}$	302
$T_{w1}$	0.0425 s	$b_{22}$	1252.8
$T_{w2}$	0.6 s	$b_{23}$	0.01
R	0.417 Hz/pu MW		
B	0.425 pu MW/Hz

The hybrid power system governor $\left( {G_{g} \left( s \right)} \right)$, the non-reheat steam turbine $\left( {G_{t} \left( s \right)} \right)$, and power system (generator and load) $\left( {G_{p} \left( s \right)} \right)$ mathematical models used in the simulations in Laplace domain are described respectively by:

10 $$G_{g} \left( s \right) = \frac{1}{{1 + T_{g} s}},$$

11 $$G_{t} \left( s \right) = \frac{1}{{1 + T_{t} s}},$$

12 $$G_{p} \left( s \right) = \frac{{K_{l} }}{{1 + T_{l} s}},$$

where $T_{g}$, $T_{t}$, and $T_{l}$ are respectively the governor, non-reheat steam turbine, and power system (generator and load) time constants.

At Area 1, a PV plant is connected to the grid via a DC chopper and a grid side inverter; the DC chopper is used to achieve maximum power point tracking (MPPT). The PV plant power $\left( {G_{PV} \left( s \right)} \right)$ is modelled as:

13 $$G_{PV} \left( s \right) = \frac{{K_{PV} }}{{1 + T_{PV} s}},$$

where $K_{PV}$ and $T_{PV}$ represent the gain and time constant of PV system with MPPT, respectively.

At area 2, a wind turbine is linked to the grid and its output power $\left( {G_{w} \left( s \right)} \right)$ is represented by a doubly fed induction generator (DFIG) driven transfer function as^39^:

14 $$G_{w} \left( s \right) = \frac{{K_{w1} }}{{1 + T_{w1} s}} \times \frac{{K_{w2} \left( {1 + T_{w2} s} \right)}}{1 + s} \times \frac{{K_{w3} }}{1 + s},$$

where $K_{w1}$, $K_{w2}$, and $K_{w3}$ and $T_{w1}$ and $T_{w2}$ represent the wind turbine gains and time constants.

The mathematical model capturing the system dynamics is providing an actual power system considering the classical power system generators and two RES generators. Input disturbances such as rapid load variations, wind speed variations, and irradiance variations were taken into account, and the frequency variations in two areas were regulated accordingly. Frequency control is typically achieved by predicting control signals and anticipated outputs, i.e., control actions and frequency variations to regulate the controlled units. The PIDA configuration presented in Fig. 4 for area 1 is mathematically rearranged by Eqs. (6) and (7) to have tuning parameters $(a_{10} , a_{11} , a_{12} , a_{13} , b_{10} , b_{11} , b_{12} , b_{13}$), and similarly of PIDA controller in area 2 with tuning parameters $(a_{20} , a_{21} , a_{22} , a_{23} , b_{20} , b_{21} , b_{22} ,b_{23}$). The following equations are derived based on the dynamic characteristics of power and frequency variations in the two-area hybrid power system model:

15 $$D_{t} \Delta F_{1} \left( t \right) = \frac{{K_{l} }}{{T_{l} }}\left[ {\Delta P_{m1} \left( t \right) + \Delta P_{PV} \left( t \right) - \Delta P_{tie} \left( t \right) - \Delta P_{L1} \left( t \right) - \frac{1}{{K_{l} }}\Delta F_{1} \left( t \right)} \right],$$

16 $$D_{t} \Delta F_{2} \left( t \right) = \frac{{K_{l} }}{{T_{l} }}\left[ {\Delta P_{m2} \left( t \right) + \Delta P_{w} \left( t \right) + \Delta P_{tie} \left( t \right) - \Delta P_{L2} \left( t \right) - \frac{1}{{K_{l} }}\Delta F_{2} \left( t \right)} \right],$$

17 $$D_{t} \theta_{1} \left( t \right) = \gamma \Delta F_{1\;ref} \left[ {\Delta F_{1} \left( t \right) - \Delta F_{1\;ref} } \right],$$

18 $$D_{t} \theta_{2} \left( t \right) = \gamma \Delta F_{2\;ref} \left[ {\Delta F_{2} \left( t \right) - \Delta F_{2\;ref} } \right],$$

19 $$D_{t} \Delta P_{v1} \left( t \right) = \frac{1}{{T_{g} }}\left[ {\Delta P_{1\;ref} \left( t \right) - \frac{1}{R}\Delta F_{1} \left( t \right) - \Delta P_{v1} \left( t \right)} \right],$$

20 $$D_{t} \Delta P_{m1} \left( t \right) = \frac{1}{{T_{t} }}\left[ {\Delta P_{v1} \left( t \right) - \Delta P_{m1} \left( t \right)} \right],$$

21 $$D_{t} \Delta P_{m2} \left( t \right) = \frac{1}{{T_{t} }}\left[ {\Delta P_{v2} \left( t \right) - \Delta P_{m2} \left( t \right)} \right],$$

22 $$D_{t} \Delta P_{v2} \left( t \right) = \frac{1}{{T_{g} }}\left[ {\Delta P_{2\;ref} \left( t \right) - \frac{1}{R}\Delta F_{2} \left( t \right) - \Delta P_{v2} \left( t \right)} \right],$$

23 $$\begin{aligned} D_{t}^{\lambda + 1} U_{1} \left( t \right) & = \gamma \Delta F_{1\;ref} \left( {k_{p} D_{t}^{\lambda } + k_{i} + k_{d} D_{t}^{\mu + \lambda } } \right) \\ & \quad \times \left[ {\Delta F_{1\;ref} \Delta F_{1} \left( t \right) + \mathop \smallint \limits_{0}^{t} \Delta F_{1} \left( \tau \right)ACE_{1} \left( {t - \tau } \right)d\tau - \Delta F_{1\;ref} ACE_{1} \left( t \right) - \mathop \smallint \limits_{0}^{t} \Delta F_{1} \left( \tau \right)\Delta F_{1} \left( {t - \tau } \right)d\tau } \right], \\ \end{aligned}$$

24 $$\begin{aligned} D_{t}^{\lambda + 1} U_{2} \left( t \right) & = \gamma \Delta F_{2\;ref} \left( {k_{p} D_{t}^{\lambda } + k_{i} + k_{d} D_{t}^{\mu + \lambda } } \right) \\ & \quad \times \left[ {\Delta F_{2\;ref} \Delta F_{2} \left( t \right) + \mathop \smallint \limits_{0}^{t} \Delta F_{2} \left( \tau \right)ACE_{2} \left( {t - \tau } \right)d\tau - \Delta F_{2\;ref} ACE_{2} \left( t \right) - \mathop \smallint \limits_{0}^{t} \Delta F_{2} \left( \tau \right)\Delta F_{2} \left( {t - \tau } \right)d\tau } \right], \\ \end{aligned}$$

25 $$\begin{aligned} & \left( {T_{{w_{2} }} D_{t}^{3} + \left( {2T_{{w_{2} }} + 1} \right)D_{t}^{2} + \left( {T_{{w_{2} }} + 2} \right)D_{t} + 1} \right)\Delta P_{w} \left( t \right) = k_{{w_{1} }} k_{{w_{2} }} k_{{w_{3} }} \left( {1 + T_{{w_{2} }} D_{t} } \right) \\ & \quad \times \left[ {\mathop \smallint \limits_{0}^{t} V_{w} \left( \tau \right)\Delta P_{2\;ref} \left( {t - \tau } \right)d\tau - \frac{1}{R}\mathop \smallint \limits_{0}^{t} \Delta P_{2\;ref} \left( \tau \right)\Delta F_{2} \left( {t - \tau } \right)d\tau } \right], \\ \end{aligned}$$

26 $$\left( {b_{10} D_{t}^{3} + b_{11} D_{t}^{2} + b_{12} D_{t} + b_{13} } \right)\Delta P_{1\;ref} \left( t \right) = \left( {a_{10} D_{t}^{3} + a_{11} D_{t}^{2} + a_{12} D_{t} + a_{13} } \right)U_{1} \left( t \right),$$

27 $$\left( {b_{20} D_{t}^{3} + b_{21} D_{t}^{2} + b_{22} D_{t} + b_{23} } \right)\Delta P_{2\;ref} \left( t \right) = \left( {a_{20} D_{t}^{3} + a_{21} D_{t}^{2} + a_{22} D_{t} + a_{23} } \right)U_{2} \left( t \right),$$

28 $$D_{t} \Delta P_{pv} \left( t \right) = \frac{{k_{pv} }}{{T_{pv} }}\left[ {\mathop \smallint \limits_{0}^{t} I\left( \tau \right)\Delta P_{1\;ref} \left( {t - \tau } \right)d\tau - \frac{1}{{k_{pv} }}\Delta P_{pv} \left( t \right)} \right],$$

29 $$D_{t} \Delta P_{tie} \left( t \right) = 2\pi T_{12} \left[ {\Delta F_{1} \left( t \right) - \Delta F_{2} \left( t \right)} \right],$$

30 $$ACE_{1} \left( t \right) = - \Delta P_{tie} \left( t \right) - B\Delta F_{1} \left( t \right),$$

31 $$ACE_{2} \left( t \right) = \Delta P_{tie} \left( t \right) - B\Delta F_{2} \left( t \right).$$

where $U\left( t \right)$ serves as the FOAPID controller’s output directed to the HS-PIDA controller. $K_{p}$, $K_{i}$, and $K_{d}$ represent the proportional, integral, and derivative coefficients of the FOAPID controller, respectively. $\lambda$ and $\mu$ represent the fractional orders associated with the integrator and differentiator, respectively. $\theta \left( t \right)$ is the output of the adaptive mechanism in the FOAPID controller, $\gamma$ is the learning rate of the adaptive mechanism, and $\Delta F_{ref1}$ and $\Delta F_{ref2}$ represent the target frequency deviation values for both areas and are predefined as a constant value tends to zero. $\Delta P_{ref} \left( t \right)$ is the reference set power of the governor. $\Delta F_{1} \left( t \right)$ and $\Delta F_{2} \left( t \right)$ are the frequency variations of area 1 and area 2, respectively. $\Delta P_{tie} \left( t \right)$ is the tie line power flow variation. $\Delta P_{m1} \left( t \right)$ and $\Delta P_{m2} \left( t \right)$ are the changes in the turbine mechanical power in area 1 and area 2, respectively. $\Delta P_{PV} \left( t \right)$ is the PV power change and $\Delta P_{w} \left( t \right)$ is the wind power change. $\Delta P_{L1} \left( t \right)$ and $\Delta P_{L2} \left( t \right)$ refer to the load disturbances affecting area 1 and area 2, respectively. $I\left( t \right)$ is the solar irradiance. $R$ is the speed regulation and $B$ is the frequency bias factor. $T_{12}$ is the tie line synchronizing coefficient.

## Results and discussions

Six case studies are presented within the simulation results for the proposed cascaded controller robustness check. In the first case, the proposed controller is compared to Fuzzy PID, TLBO PIDA, and SCA PIDA controllers when there is a step load perturbation equals to 0.01 per unit (pu) in area 1. At the second case, a predefined dynamic disturbance in area 1 is applied and the proposed controller response is evaluated against the previously mentioned controllers at case one. In the third case it is required to test our controller in presence of PV and wind uncertainties, so the proposed controller is evaluated against controllers reported in the literature but when there is a step load perturbation equals to 0.2 pu in the hybrid system’s area 1 and area 2 to test the proposed controller’s robustness and reliability. In the fourth case, a robustness test is performed when there are irregular PV and wind fluctuations in addition to a step load disturbance equals to 0.2 pu in the hybrid system’s area 1 and area 2. After that, in the fifth case, the proposed controller is compared with Marine Predator Algorithm (MPA)-Cascaded PIDA. Finally, in the last case study, the proposed controller is examined against parameters’ uncertainty. Furthermore, a statistical analysis is executed in each case to indicate the superior performance of the proposed controller against other controllers.

### Case study 1

**Fig. 5:**
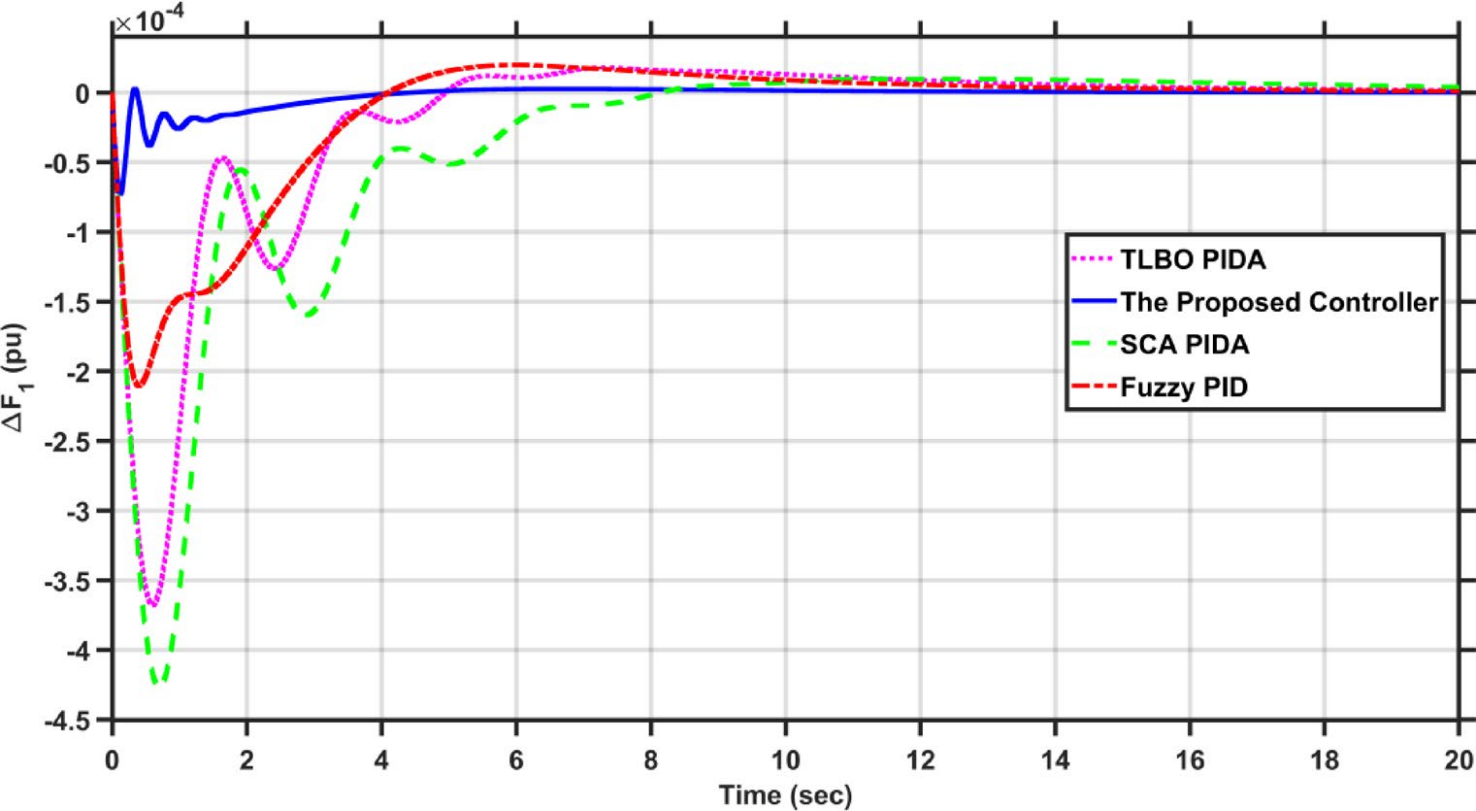
$\Delta F_{1}$ under a step disturbance equals to 0.01 pu at area 1.

**Fig. 6:**
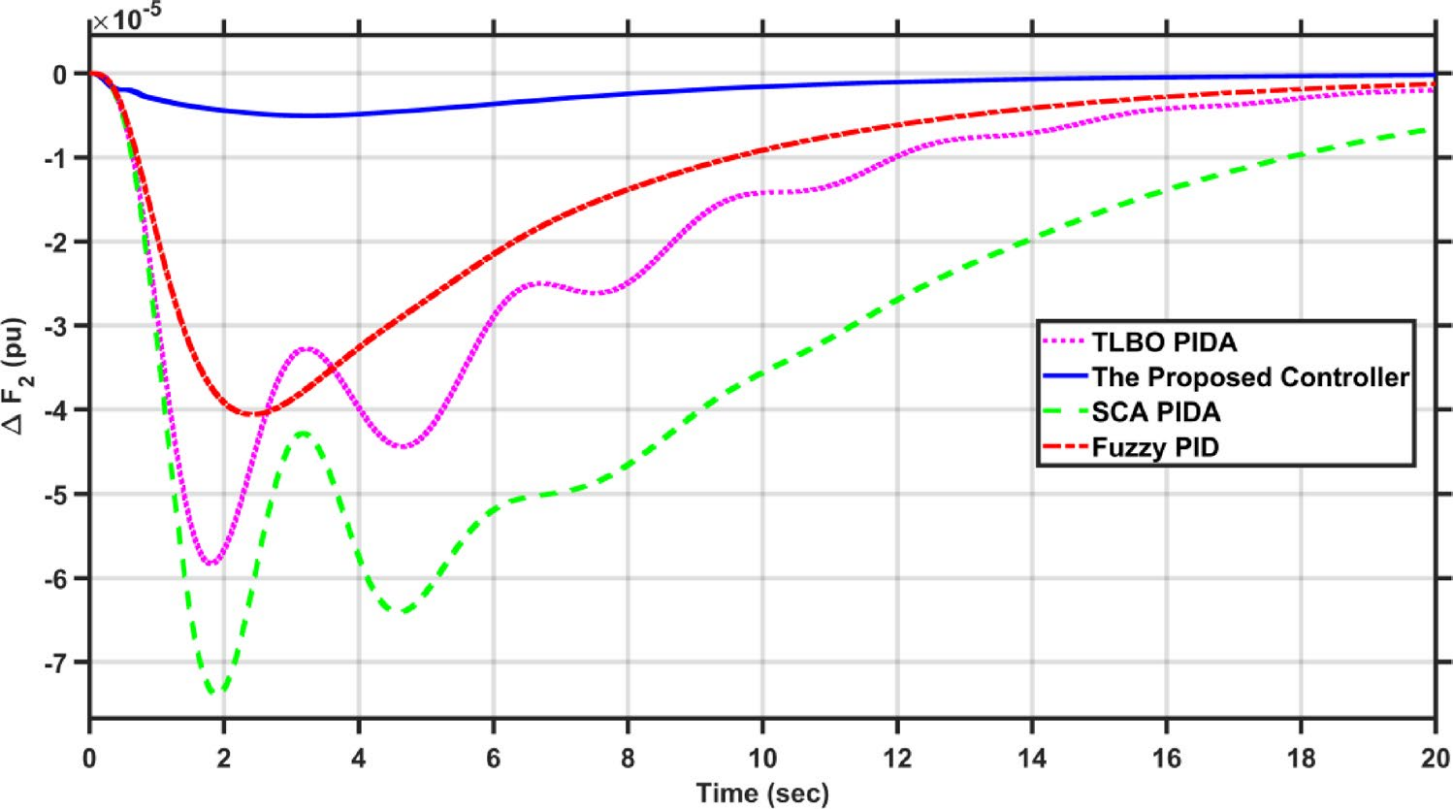
$\Delta F_{2}$ under a step disturbance equals to 0.01 pu at area 1.

**Fig. 7:**
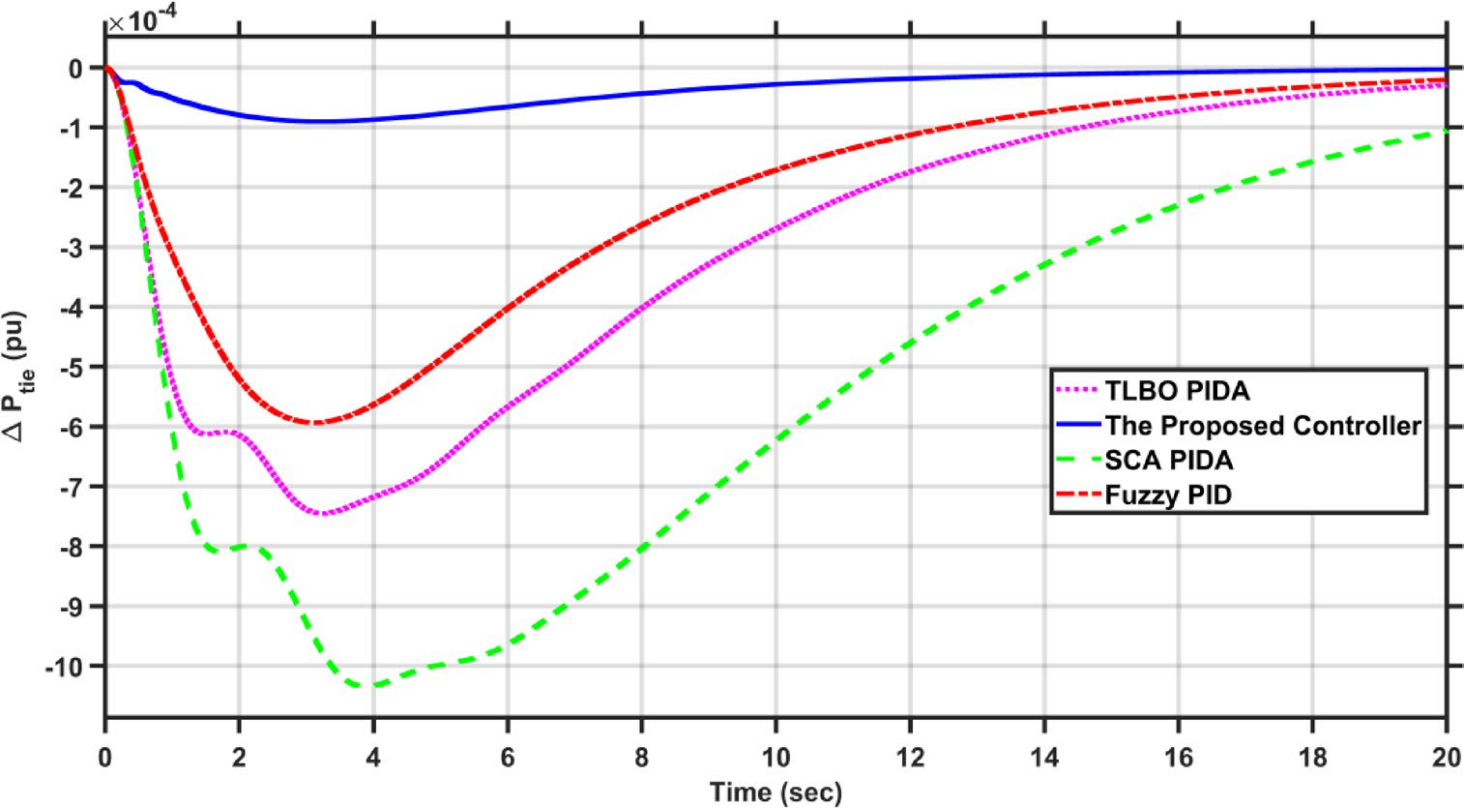
$\Delta P_{tie}$ under a step disturbance equals to 0.01 pu at area 1.

In this case, the first area of the conventional system experiences a 1% increase in its load. The frequency deviations across the two areas, along with the tie-line power deviation, are depicted in Figs. 5, 6 and 7, respectively. Clearly, the proposed controller outperforms the other controllers, as the settling time and undershoot are significantly decreased. Table 2 presents a comparison of transient stability indicators between the proposed controller and other controllers. The results clearly indicate that the proposed controller has the minimum settling time for $\Delta F_{1}$ which is 3.1065 s, while it is 14.0564 s, 17.9242 s, and 11.9014 s for TLBO PIDA, SCA PIDA, and Fuzzy PID, respectively. Also, our controller was the only one that reached the steady state for $\Delta F_{2}$ and $\Delta P_{tie}$ with a settling time of 14.866 s and 17.669 s, respectively, while the other controllers have a steady state error. In addition, the proposed controller has an undershoot that is 80.42% lower than the TLBO PIDA, 82.75% lower than the SCA PIDA, and 63.78% lower than the Fuzzy PID for $\Delta F_{1}$ and for $\Delta F_{2}$ the undershoot of the proposed controller is 91.36% lower than the TLBO PIDA, 93.18% lower than the SCA PIDA, and 87.6% lower than the Fuzzy PID. Moreover, the undershoot of the proposed controller is 87.97% lower than the TLBO PIDA, 90.98% lower than the SCA PIDA, and 84.96% lower than the Fuzzy PID for $\Delta P_{tie}$.

**Table 2:** Transient stability indicators of case 1.

	Controller	Undershoot (pu)	Settling time (s)
$\Delta F_{1}$	The proposed controller	− 7.2451$e^{ - 5}$	3.1065
	TLBO PIDA	− 0.00037	14.0564
	SCA PIDA	− 0.00042	17.9242
	Fuzzy PID	− 0.0002	11.9014
$\Delta F_{2}$	The proposed controller	− 5.0332$e^{ - 6}$	14.866
	TLBO PIDA	− 5.8246$e^{ - 5}$	–
	SCA PIDA	− 7.3838$e^{ - 5}$	–
	Fuzzy PID	− 4.0589$e^{ - 5}$	–
$\Delta P_{tie}$	The proposed controller	− 9.0217$e^{ - 5}$	17.669
	TLBO PIDA	− 0.00075	–
	SCA PIDA	− 0.001	–
	Fuzzy PID	− 0.0006	–

Table 3 presents a comparison of the proposed controller and other controllers based on the sum of squares error (SSE) and root mean square error (RMSE). Clearly, the proposed controller has the lowest SSE and RMSE against the other controllers for $\Delta F_{1}$, $\Delta F_{2}$, and $\Delta P_{tie}$, respectively. In case of $\Delta F_{1}$, the proposed controller has a SSE that is 98.56%, 99.12%, and 97.16% lower than TLBO PIDA, SCA PIDA, and Fuzzy PID, respectively. While for $\Delta F_{2}$, the proposed controller has a SSE that is 98.84%, 99.53%, and 97.92% lower than TLBO PIDA, SCA PIDA, and Fuzzy PID, respectively. And for $\Delta P_{tie}$, the proposed controller has a SSE that is 98.62%, 99.48%, and 97.38% lower than TLBO PIDA, SCA PIDA, and Fuzzy PID, respectively. While for RMSE, the proposed controller is 87.89%, 90.55%, and 83.17% lower than TLBO PIDA, SCA PIDA, and Fuzzy PID, respectively for $\Delta F_{1}$ and for $\Delta F_{2}$ the RMSE is 89.47%, 93.24%, and 85.71% lower than TLBO PIDA, SCA PIDA, and Fuzzy PID, respectively. Finally, for $\Delta P_{tie}$ the proposed controller has a RMSE that is 88.35%, 92.74%, and 84.28% lower than TLBO PIDA, SCA PIDA, and Fuzzy PID, respectively.

**Table 3:** Statistical analysis of case 1.

	Controller	SSE	RMSE
$\Delta F_{1}$	The proposed controller	4.737$e^{ - 7}$	0.00069
	TLBO PIDA	3.2862$e^{ - 5}$	0.0057
	SCA PIDA	5.3965$e^{ - 5}$	0.0073
	Fuzzy PID	1.6668$e^{ - 5}$	0.0041
$\Delta F_{2}$	The proposed controller	4.085$e^{ - 8}$	0.0002
	TLBO PIDA	3.5289$e^{ - 6}$	0.0019
	SCA PIDA	8.76$e^{ - 6}$	0.00296
	Fuzzy PID	1.9669$e^{ - 6}$	0.0014
$\Delta P_{tie}$	The proposed controller	1.3094$e^{ - 5}$	0.0036
	TLBO PIDA	0.00095	0.0309
	SCA PIDA	0.0025	0.0496
	Fuzzy PID	0.0005	0.0229

### Case study 2

**Fig. 8:**
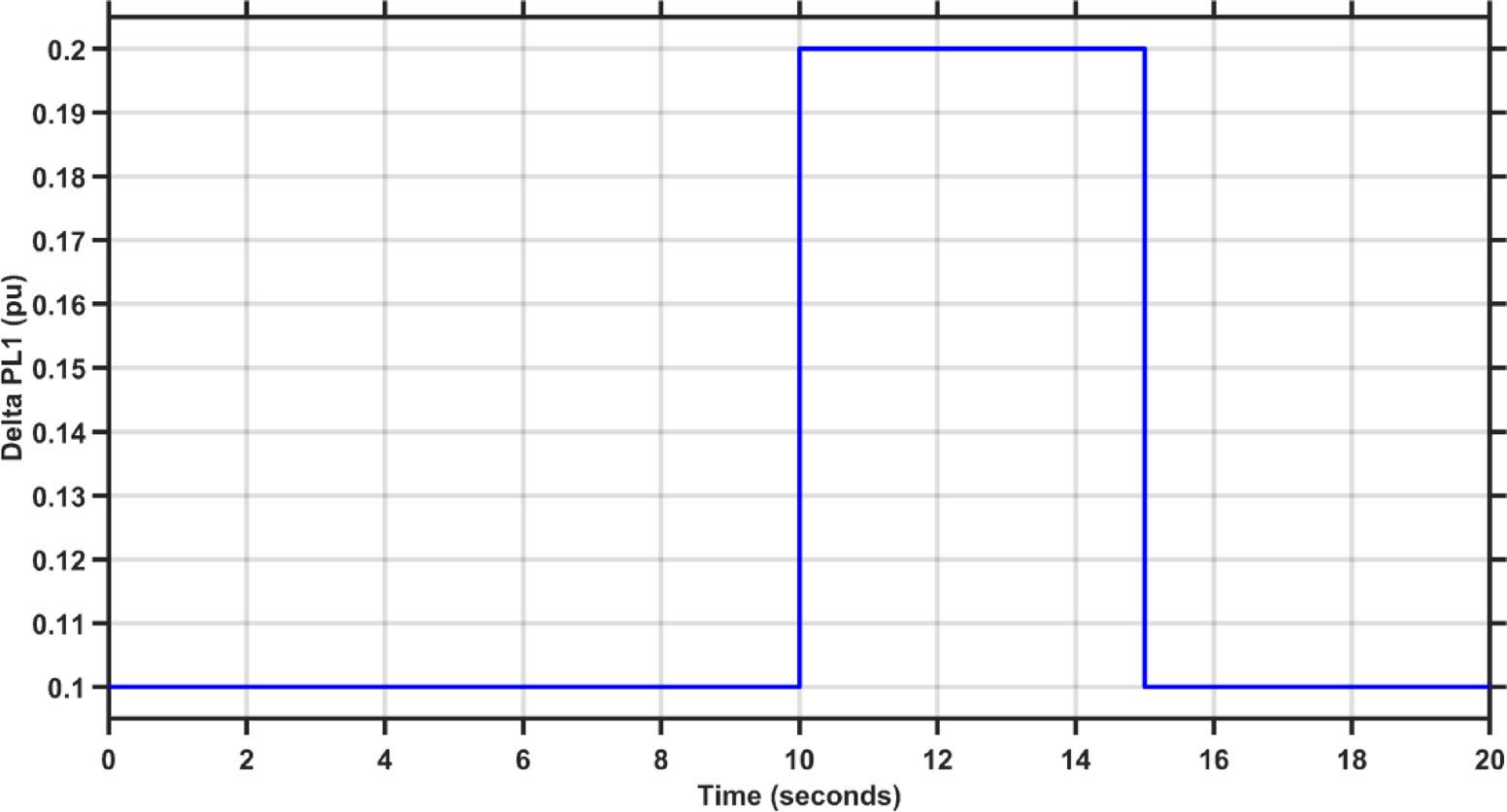
The dynamic disturbance applied to area 1.

**Fig. 9:**
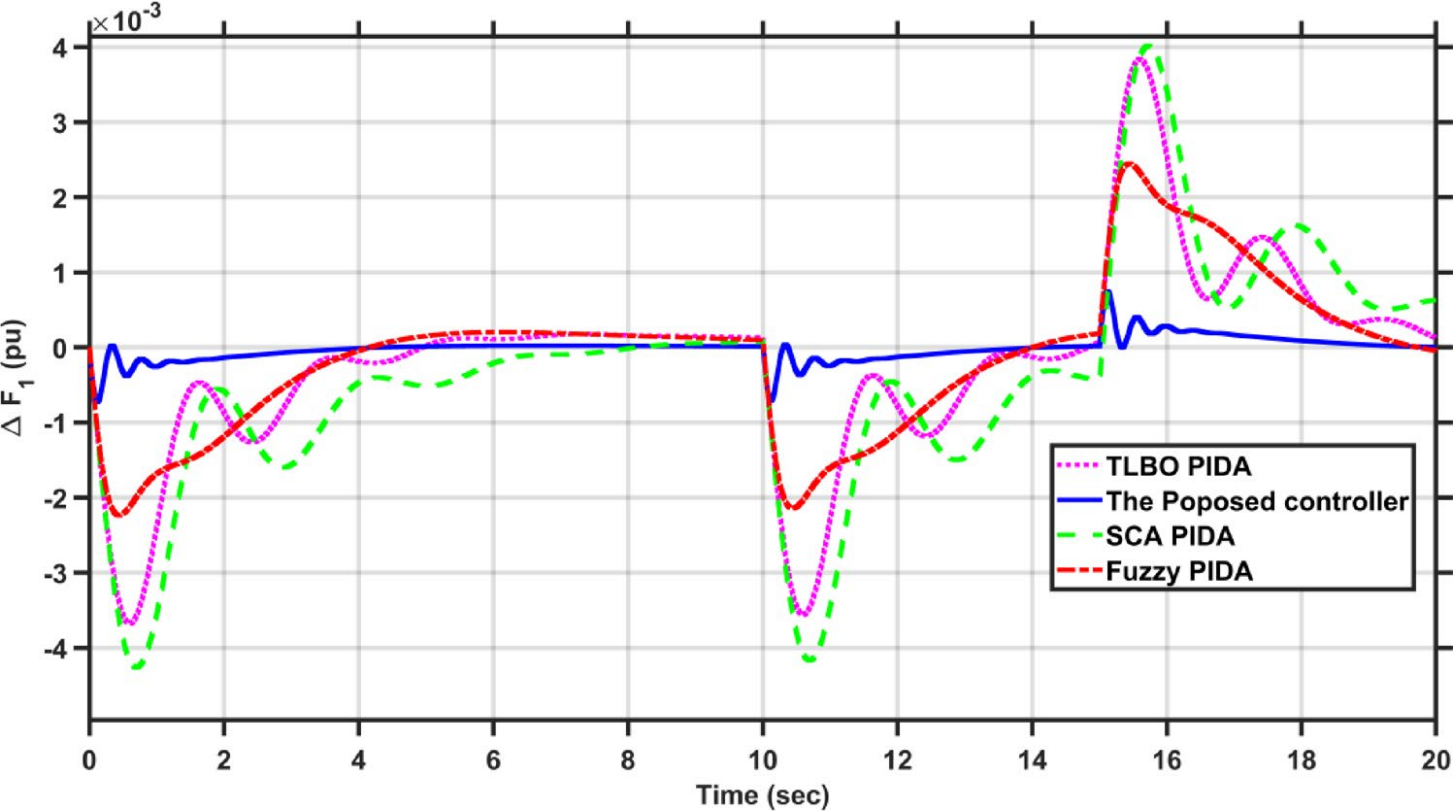
$\Delta F_{1}$ under the dynamic disturbance at area 1.

**Fig. 10:**
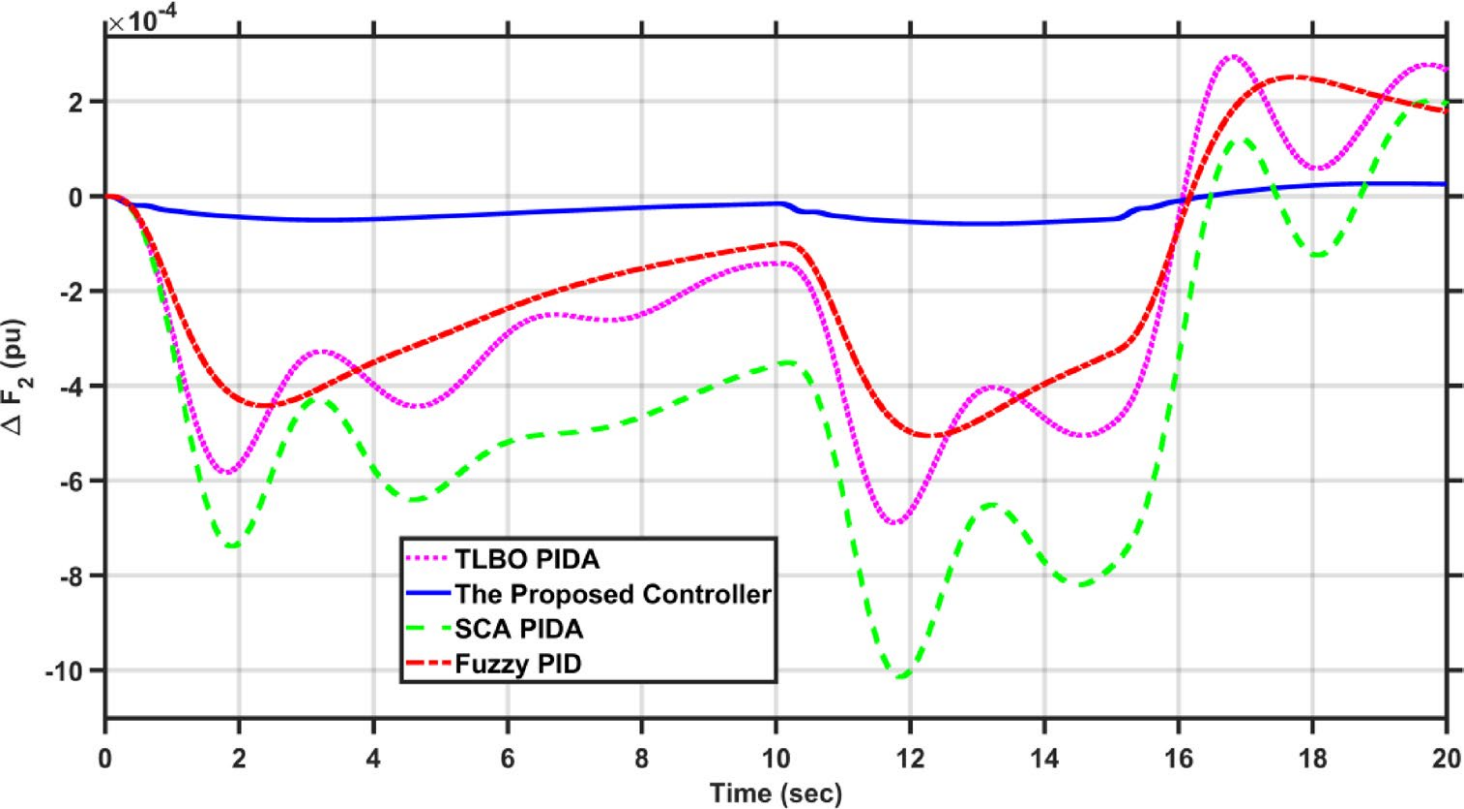
$\Delta F_{2}$ under the dynamic disturbance at area 1.

**Fig. 11:**
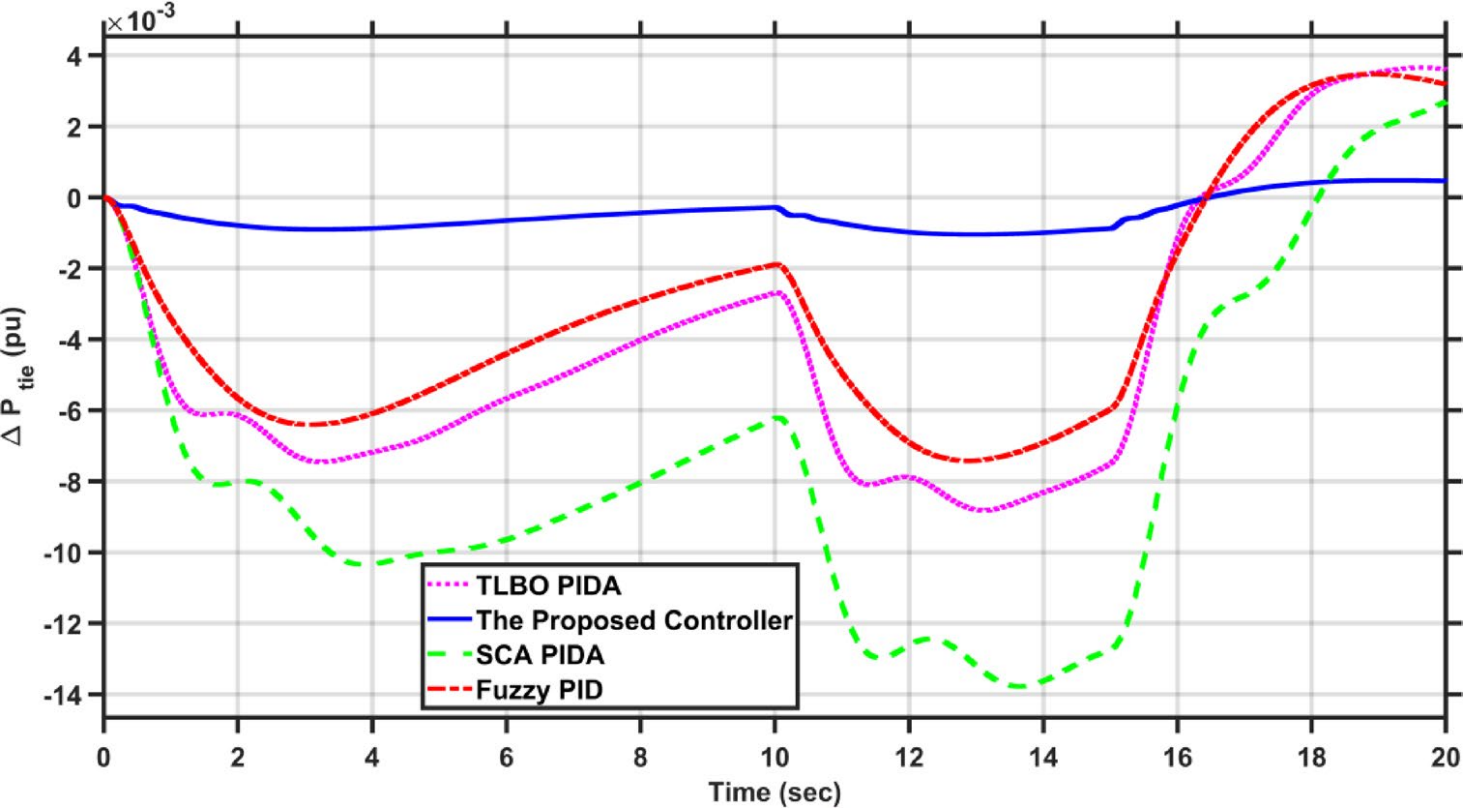
$\Delta P_{tie}$ under the dynamic disturbance at area 1.

In this case, the load of area 1 experiences a dynamic change that is depicted in Fig. 8. Figures 9, 10 and 11 show the frequency deviations in the two areas and the tie line power deviation, respectively. The results clearly show that the proposed controller provides the best dynamic response as it has the lowest undershoots and overshoots during the three step changes, and it has the least settling time, while the other controllers couldn’t settle and have steady state errors for $\Delta F_{1}$, $\Delta F_{2}$, and $\Delta P_{tie}$, respectively. The proposed controller has settling times of 3.3049 s, 13.1848s, and 18.7416 s during the three step changes for $\Delta F_{1}$. Although the proposed controller couldn’t settle for $\Delta F_{2}$, and $\Delta P_{tie}$, it was the nearest one to zero during the three step changes. Moreover, for $\Delta F_{1}$ the proposed controller undershoot and overshoot are lower than TLBO PIDA, SCA PIDA, and Fuzzy PID by (80.43–80.06–80.42%), (83.1–82.97–81.3%), and (67.86–66.82–69.26%) during the three step changes, respectively. While for $\Delta F_{2}$, the undershoots and overshoot of the proposed controller are lower than TLBO PIDA, SCA PIDA, and Fuzzy PID by (91.32–91.53–90.88%), (93.2–94.21–86.78%), and (88.56–88.54–89.42%) during the three step changes, respectively. Finally, for $\Delta P_{tie}$ the undershoots and overshoot of the proposed controller are lower than TLBO PIDA, SCA PIDA, and Fuzzy PID by (87.92–88.08–86.89%), (91.3–92.38–82.16%), and (85.94–85.85–86.17%) during the three step changes, respectively.

Table 4 indicates the statistical analysis indices of each controller. The results indicate that the proposed controller achieves the lowest SSE and RMSE for $\Delta F_{1}$, $\Delta F_{2}$, and $\Delta P_{tie}$, respectively. For SSE, the proposed controller is lower than TLBO PIDA, SCA PIDA, and Fuzzy PID by (99–99.34–98.39%), (98.92–99.52–98.28%), and (98.58–99.42–97.9%) for $\Delta F_{1}$, $\Delta F_{2}$, and $\Delta P_{tie}$, respectively. While for RMSE, the proposed controller is lower than TLBO PIDA, SCA PIDA, and Fuzzy PID by (87.9–90.16–84.61%), (89.57–93.1–87.39%), and (88.17–92.42–85.64%) for $\Delta F_{1}$, $\Delta F_{2}$, and $\Delta P_{tie}$, respectively.

**Table 4:** Statistical analysis of case 2.

	Controller	SSE	RMSE
$\Delta F_{1}$	The proposed controller	0.0001	0.0121
	TLBO PIDA	0.01	0.1
	SCA PIDA	0.0151	0.122997
	Fuzzy PID	0.0062	0.0786
$\Delta F_{2}$	The proposed controller	8.609$e^{ - 6}$	0.0029
	TLBO PIDA	0.0008	0.0278
	SCA PIDA	0.0018	0.042
	Fuzzy PID	0.0005	0.023
$\Delta P_{tie}$	The proposed controller	0.0028	0.0525
	TLBO PIDA	0.1969	0.4438
	SCA PIDA	0.4801	0.6929
	Fuzzy PID	0.1336	0.3655

### Case study 3

**Fig. 12:**
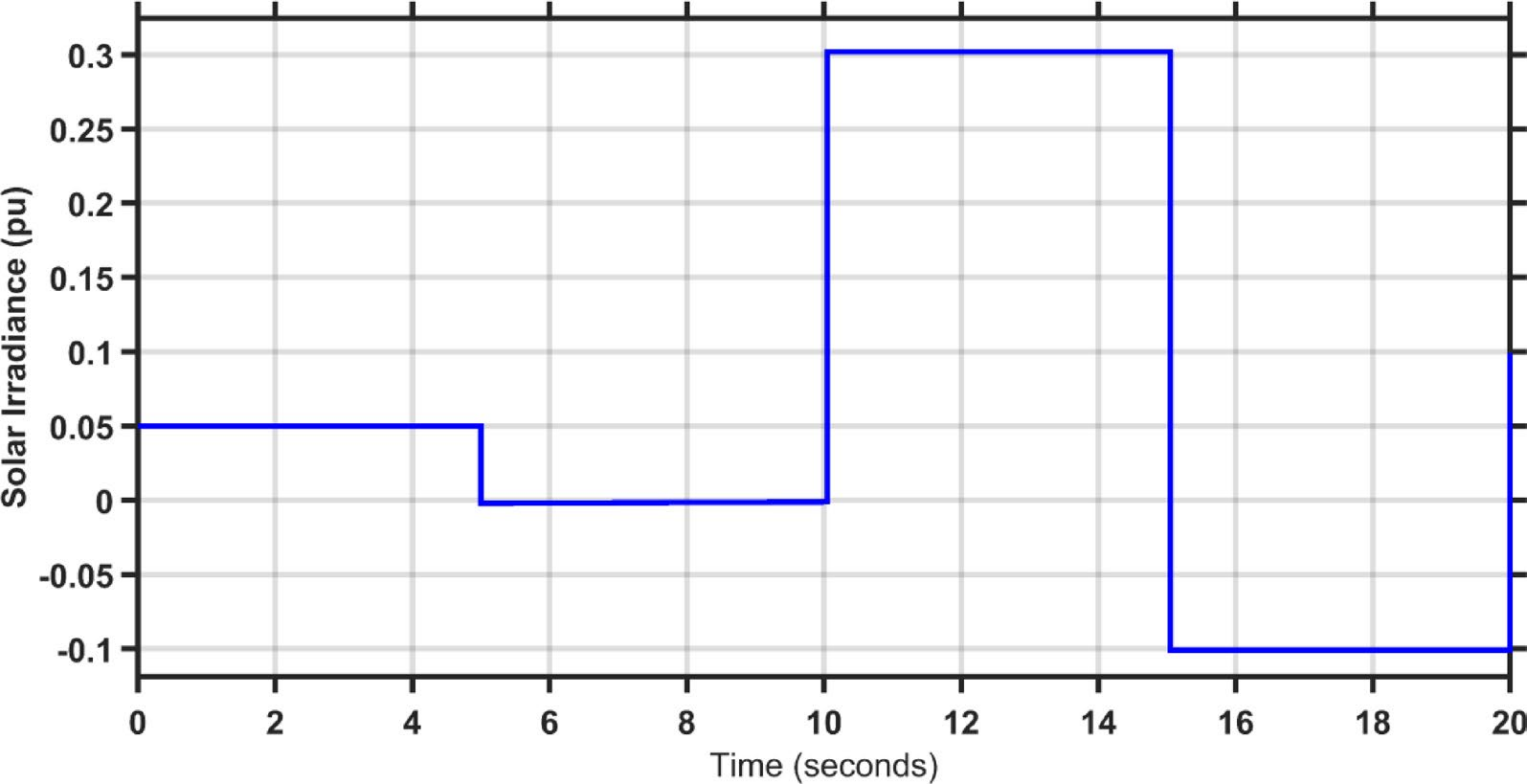
The solar irradiance fluctuations in area 1.

**Fig. 13:**
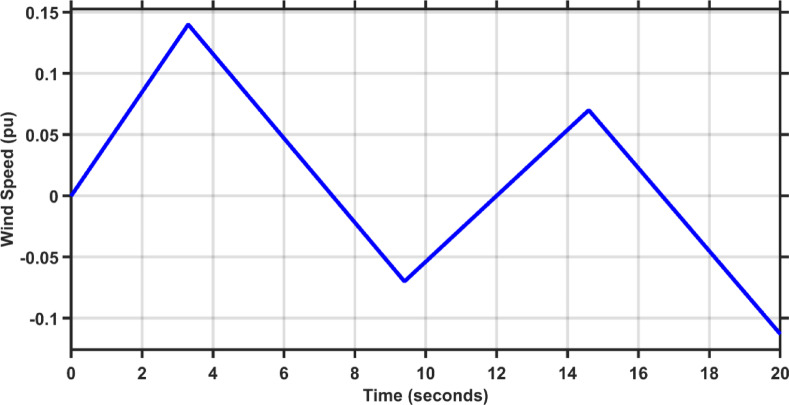
The wind speed fluctuations in area 2.

**Fig. 14:**
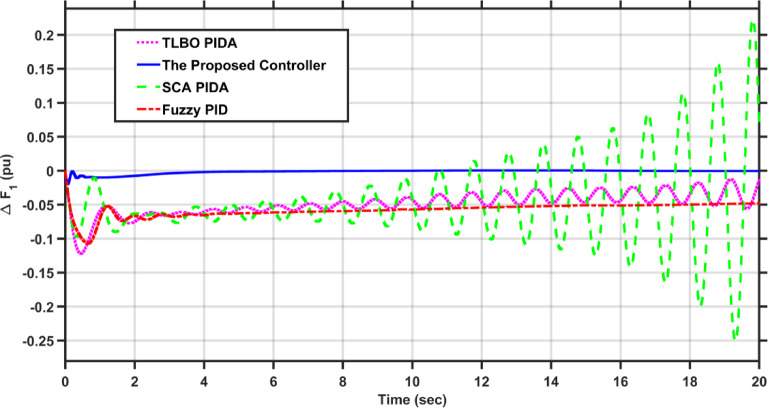
$\Delta F_{1}$ under a step disturbance equals to 0.2 pu at area 1 and area 2.

**Fig. 15:**
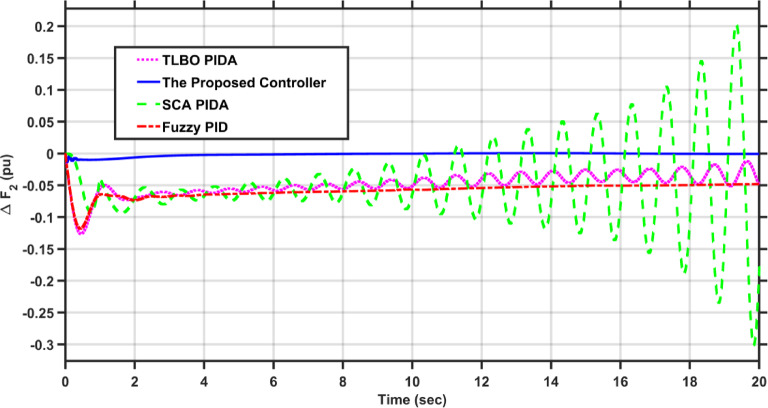
$\Delta F_{2}$ under a step disturbance equals to 0. 2 pu at area 1 and area 2.

**Fig. 16:**
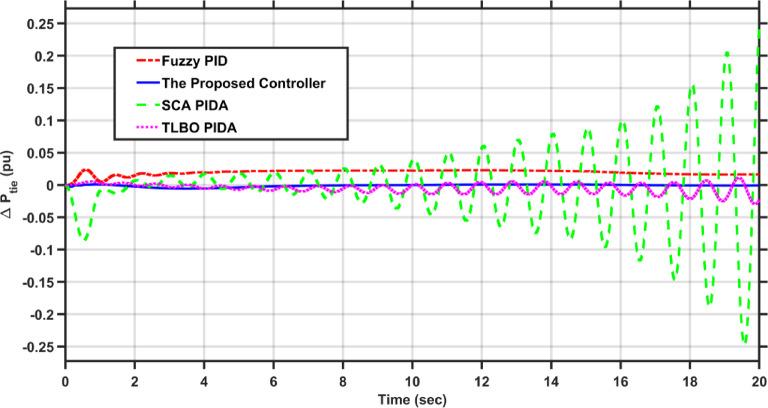
$\Delta P_{tie}$ under a step disturbance equals to 0.2 pu at area 1 and area 2.

This scenario evaluates the performance of the proposed controller against TLBO PIDA, SCA PIDA, and Fuzzy PID when increasing the load of the hybrid power system in both areas by 20%. The fluctuations in PV and wind speed are shown in Figs. 12 and 13, respectively. Figures 14, 15 and 16 depict the frequency variations of both areas and the tie-line power variation, respectively. The results prove the superiority of the proposed controller over TLBO PIDA, SCA PIDA, and Fuzzy PID, as TLBO PIDA, SCA PIDA went unstable while Fuzzy PID have steady state errors for $\Delta F_{1}$, $\Delta F_{2}$, and $\Delta P_{tie}$, respectively. The proposed controller has settlings time of 3.7371 s, 3.1547 s, and 5.8584 s while Fuzzy PID has steady state errors of − 0.0479 pu, − 0.048 pu, and 0.0163 pu for ∆F_1_, ∆F_2_, and ∆P_tie_, respectively. Moreover, the proposed controller has undershoots and overshoot that are lower than Fuzzy PID by 81.58%, 88.84% , and 99.87% for $\Delta F_{1}$, $\Delta F_{2}$, and $\Delta P_{tie}$, respectively. This case emphasizes the reliability of the proposed controller against heavy load disturbances.

Table 5 compares the statistical analysis indicators of each controller. For SSE, the proposed controller is lower than TLBO PIDA, SCA PIDA, and Fuzzy PID by (99.62–99.85–99.72%), (99.66–99.89–99.76%), and (95.06–99.91–99.05%) for $\Delta F_{1}$, $\Delta F_{2}$, and $\Delta P_{tie}$, respectively. While for RMSE, the proposed controller is lower than TLBO PIDA, SCA PIDA, and Fuzzy PID by (93.85–96.17–94.74%), (94.19–96.64–95.09%), and (77.78–97.03–90.24%) for $\Delta F_{1}$, $\Delta F_{2}$, and $\Delta P_{tie}$, respectively. These results show the wide difference between the proposed controller and TLBO PIDA, SCA PIDA, and Fuzzy PID as the proposed controller has the minimum SSE and RMSE by a significant margin for $\Delta F_{1}$, $\Delta F_{2}$, and $\Delta P_{tie}$, respectively.

**Table 5:** Statistical analysis of case 3.

	Controller	SSE	RMSE
$\Delta F_{1}$	The proposed controller	0.0776	0.2785
	TLBO PIDA	20.5086	4.5286
	SCA PIDA	52.9529	7.2769
	Fuzzy PID	28.0655	5.2977
$\Delta F_{2}$	The proposed controller	0.0682	0.2612
	TLBO PIDA	20.17798	4.49199
	SCA PIDA	60.3877	7.77095
	Fuzzy PID	28.2884	5.3187
$\Delta P_{tie}$	The proposed controller	0.0322	0.1795
	TLBO PIDA	0.6524	0.8077
	SCA PIDA	36.4731	6.0393
	Fuzzy PID	3.3788	1.8382

### Case study 4

**Fig. 17:**
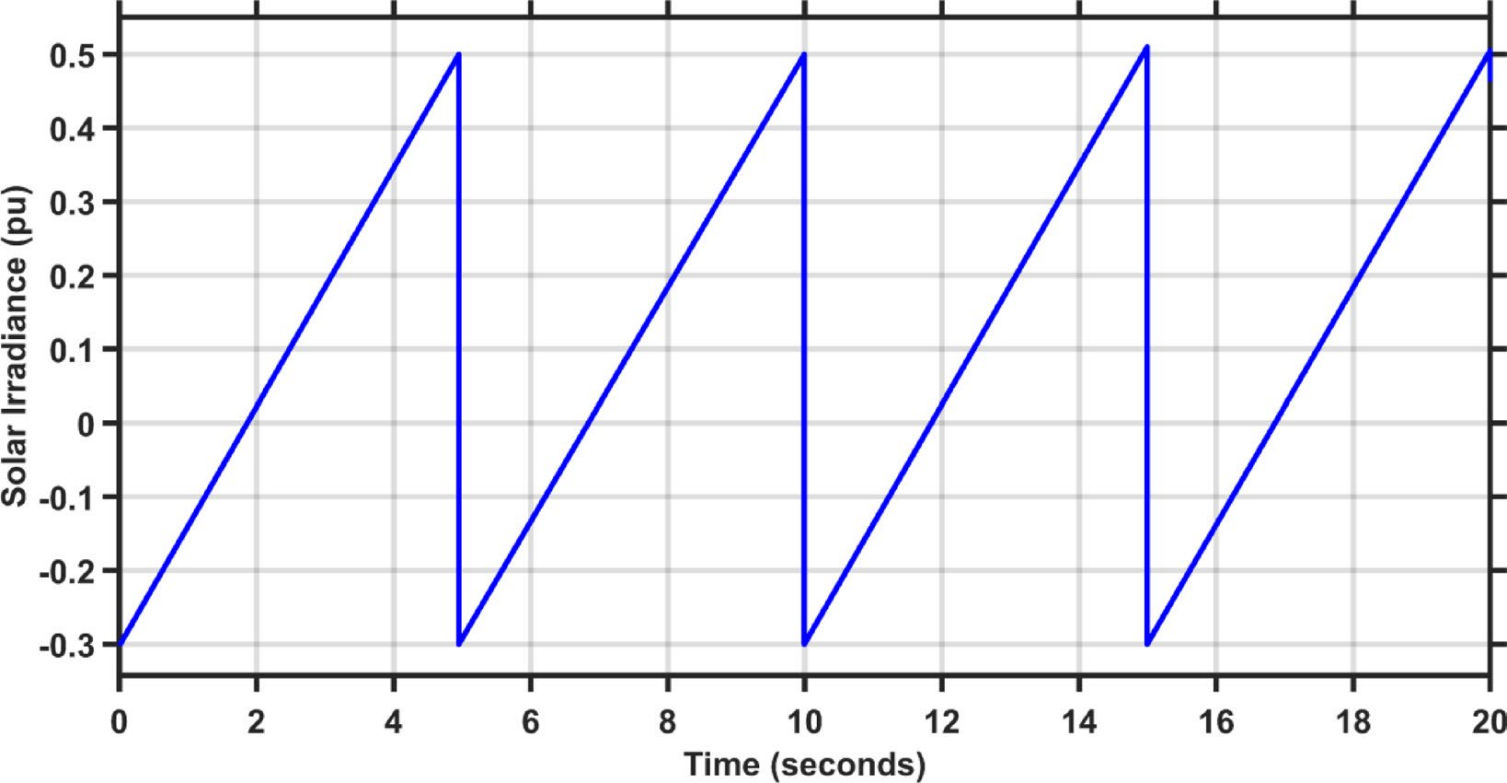
The solar irradiance fluctuations of case 4 in area 1.

**Fig. 18:**
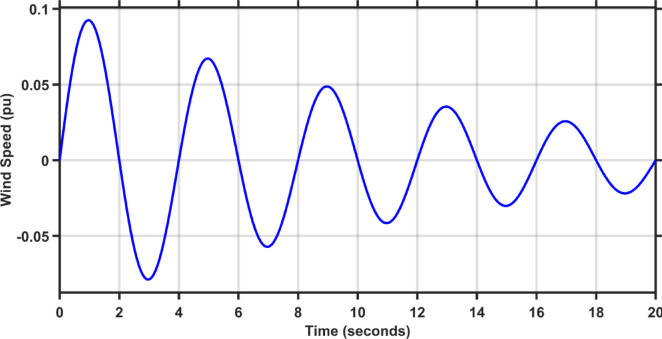
The wind speed fluctuations of case 4 in area 2.

**Fig. 19:**
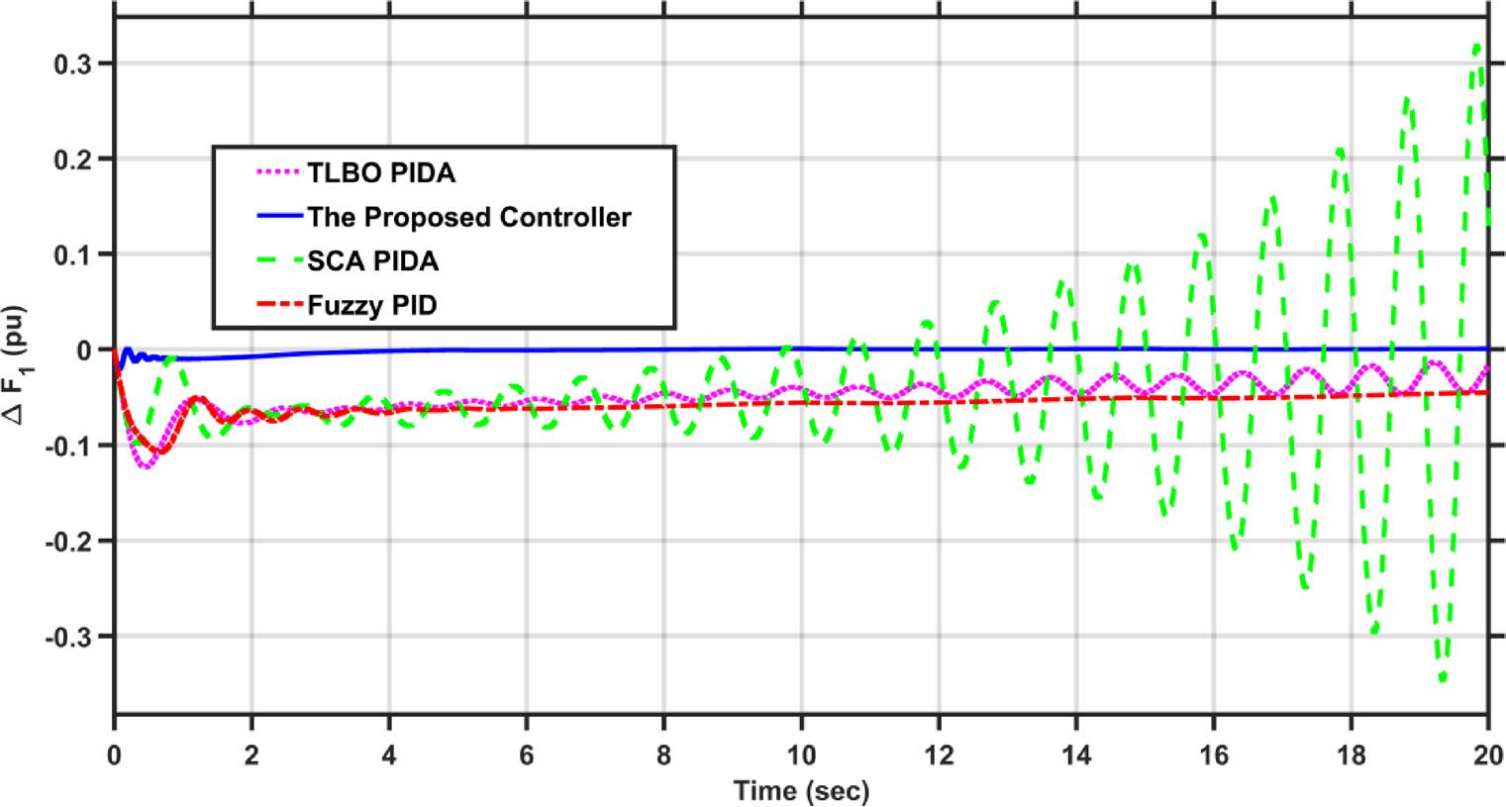
$\Delta F_{1}$ under the disturbance of case 4.

**Fig. 20:**
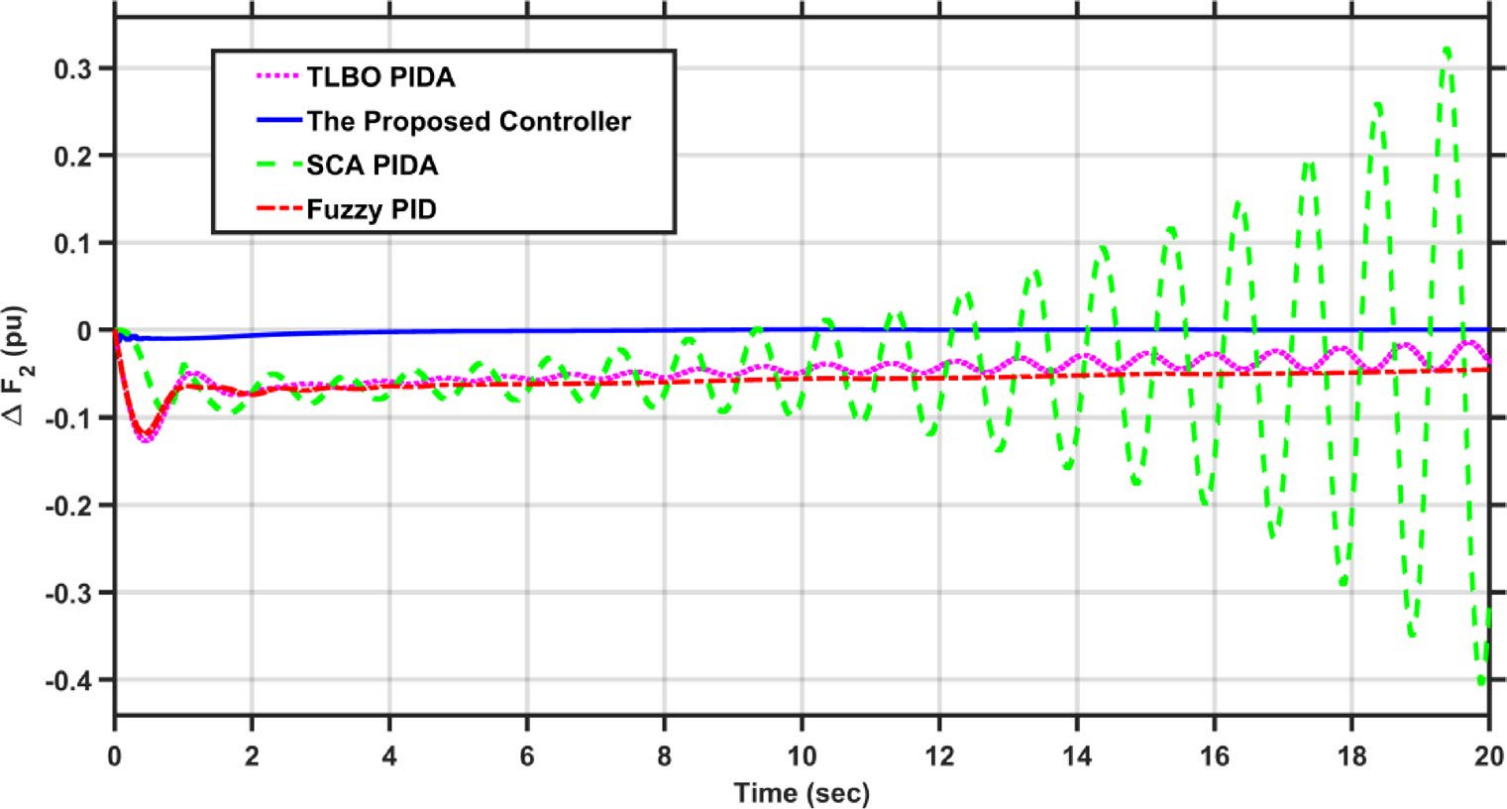
$\Delta F_{2}$ under the disturbance of case 4.

**Fig. 21:**
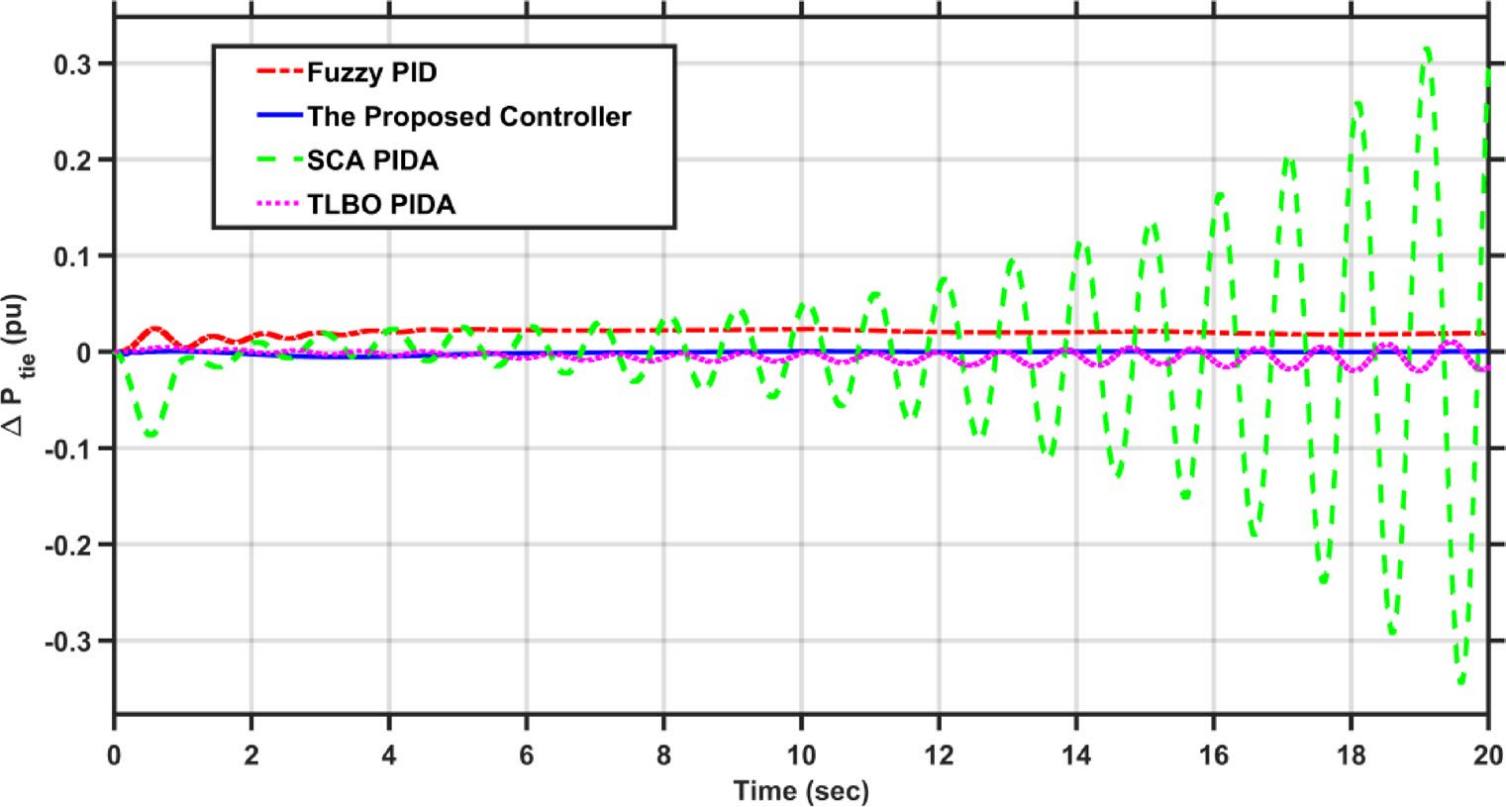
$\Delta P_{tie}$ under the disturbance of case 4.

In this scenario, the hybrid power system experiences an identical load disturbance as in case three in addition to an irregular irradiance for PV generation in area 1 and an irregular wind speed for wind generation in area 2 which are shown in Figs. 17 and 18. The frequency variations in both areas, along with the tie-line power deviation, are depicted in Figs. 19, 20 and 21 respectively. it can be noticed from the results that TLBO PIDA, SCA PIDA went unstable, the proposed controller has the least settling times and oscillations, and Fuzzy PID has a steady state error for $\Delta F_{1}$, $\Delta F_{2}$, and $\Delta P_{tie}$, respectively. The settling times of the proposed controller are 3.1021 s, 2.928 s, and 4.9299 s while the steady state errors of Fuzzy PID are − 0.0454 pu, − 0.0457 pu, and 0.0195 pu for $\Delta F_{1}$, $\Delta F_{2}$, and $\Delta P_{tie}$, respectively. In addition, the undershoots and overshoots of the proposed controller are lower than Fuzzy PID by 81%, 88.81%, and 97.9% for $\Delta F_{1}$, $\Delta F_{2}$, and $\Delta P_{tie}$, respectively. The results confirm that the proposed controller outperforms the other controllers.

Table 6 compares the SSE and RMSE of each controller. For SSE, the proposed controller is lower than TLBO PIDA, SCA PIDA, and Fuzzy PID by (99.53–99.89–99.65%), (99.59–99.92–99.69%), and (93.12–99.96–99.06%) for $\Delta F_{1}$, $\Delta F_{2}$, and $\Delta P_{tie}$, respectively. While for RMSE, the proposed controller is lower than TLBO PIDA, SCA PIDA, and Fuzzy PID by (93.16–96.68–94.05%), (93.57–97.15–94.46%), and (73.8–97.98–90.31%) for $\Delta F_{1}$, $\Delta F_{2}$, and $\Delta P_{tie}$, respectively. It is evident from the results that the proposed controller has the least values of SSE and RMSE for $\Delta F_{1}$, $\Delta F_{2}$, and $\Delta P_{tie}$, respectively.

**Table 6:** Statistical analysis of case 4.

	Controller	SSE	RMSE
$\Delta F_{1}$	The proposed controller	0.1112	0.3334
	TLBO PIDA	23.7857	4.8771
	SCA PIDA	100.6244	10.0312
	Fuzzy PID	31.3743	5.6013
$\Delta F_{2}$	The proposed controller	0.0977	0.3125
	TLBO PIDA	23.5998	4.85796
	SCA PIDA	120.3566	10.9707
	Fuzzy PID	31.7875	5.638
$\Delta P_{tie}$	The proposed controller	0.0341	0.1845
	TLBO PIDA	0.49598	0.7043
	SCA PIDA	83.6132	9.144
	Fuzzy PID	3.6257	1.9041

### Case study 5

**Fig. 22:**
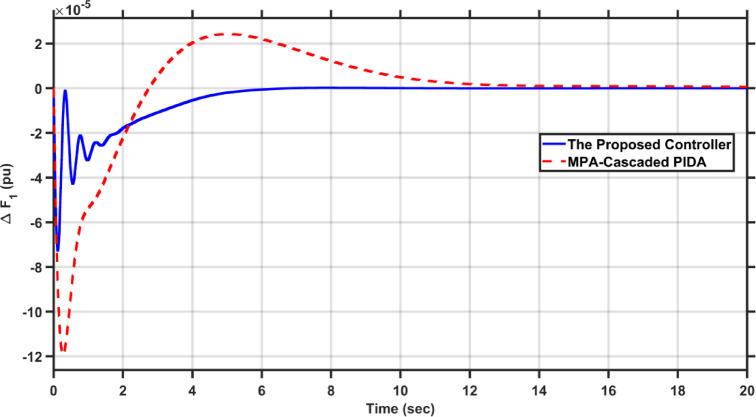
$\Delta F_{1}$ under the disturbance of case 5.

**Fig. 23:**
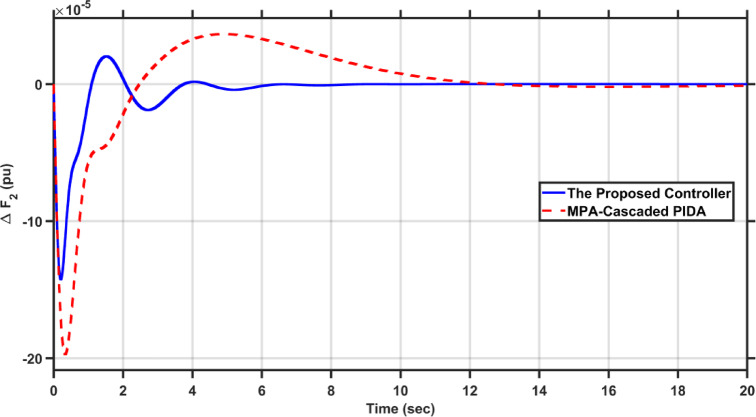
$\Delta F_{2}$ under the disturbance of case 5.

**Fig. 24:**
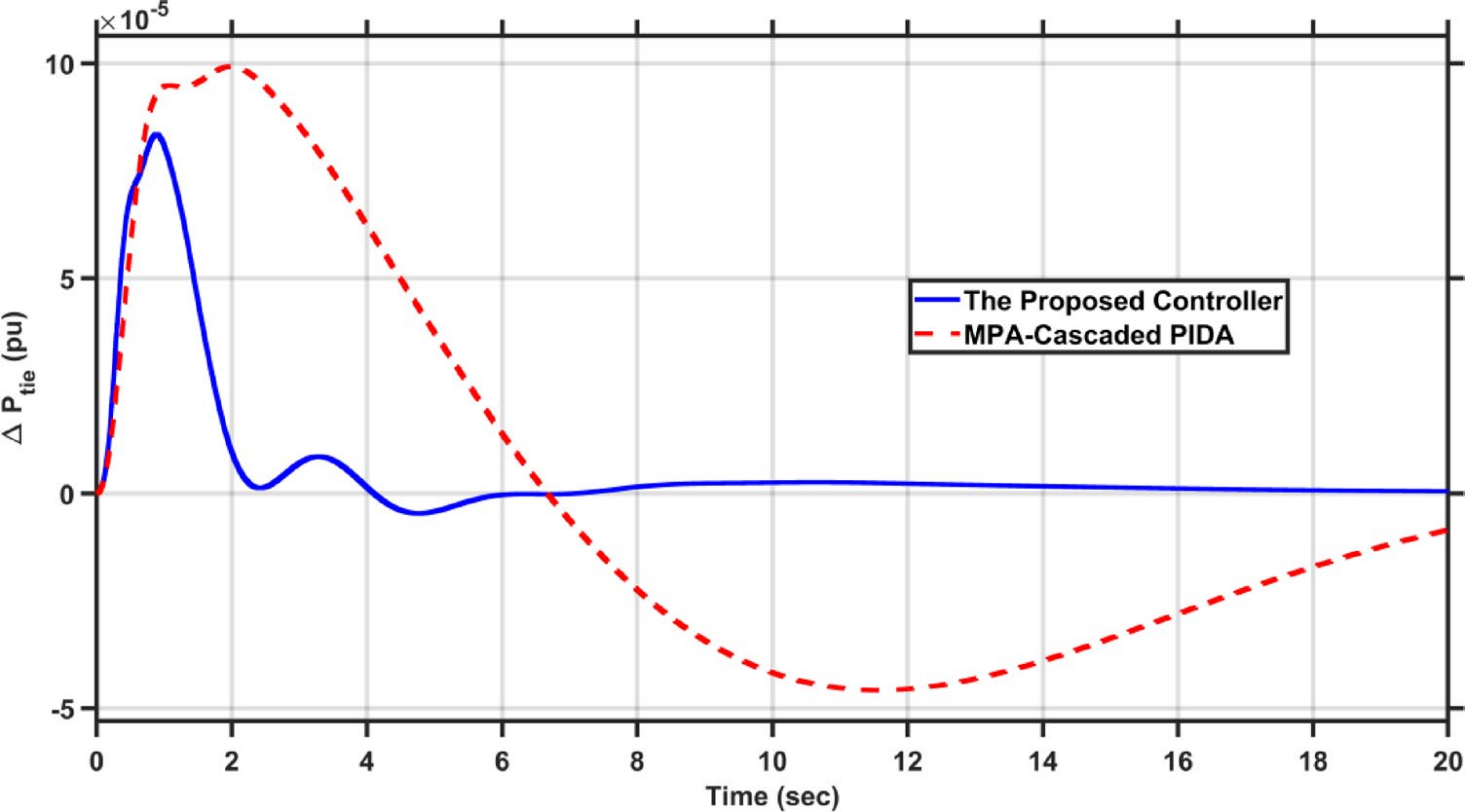
$\Delta P_{tie}$ under the disturbance of case 5.

In this scenario, the proposed controller was evaluated against the MPA-Cascaded PIDA proposed in^28^ when applying 1% step load perturbation in area 1 and area 2 of the conventional system. Figures 22, 23 and 24 presents the frequency deviations in the two areas and the tie-line power deviation, respectively.

It is clear from the results that the proposed FOAPID/HS-PIDA has a better transient response than MPA-Cascaded PIDA as it managed to decrease overshoots by 44.5%, and 15.94% for $\Delta F_{2}$, and $\Delta P_{tie}$, respectively, also it managed to decrease the undershoots by 39.42%, 28.93%, and 89.85% for $\Delta F_{1}$, $\Delta F_{2}$, and $\Delta P_{tie}$, respectively. Moreover, the proposed controller reduced the settling times of $\Delta F_{1}$, $\Delta F_{2}$, and $\Delta P_{tie}$ to 6.285 s, 6.222 s, respectively and 17.575 s instead of 10 s, 12 s, and 21 s, respectively in case of MPA-Cascaded PIDA.

### Case study 6: robustness test against parameters’ uncertainty

Furthermore, an additional analysis is carried out to assess the sensitivity of the proposed controller to variations in system parameters. Table 7 presents the overshoots (OS) and undershoots (US) for $\Delta F_{1}$, $\Delta F_{2}$, and $\Delta P_{tie}$ when changing the inertia constants of the generators ($M_{1}$ and $M_{2}$) in the two areas of the conventional system by $\pm 25\%$.

**Table 7:** Dynamic response characteristics of the proposed controller and MPA-cascaded PIDA controller for system parameters change.

	% Change	The proposed controller						MPA-cascaded PIDA					
		$\Delta F_{1}$		$\Delta F_{2}$		$\Delta P_{tie}$		$\Delta F_{1}$		$\Delta F_{2}$		$\Delta P_{tie}$	
		OS (pu)$\times 10^{ - 4}$	US (pu)$\times 10^{ - 4}$	OS (pu)$\times 10^{ - 4}$	US (pu)$\times 10^{ - 4}$	OS (pu)$\times 10^{ - 4}$	US (pu)$\times 10^{ - 4}$	OS (pu)$\times 10^{ - 4}$	US (pu)$\times 10^{ - 4}$	OS (pu)$\times 10^{ - 4}$	US (pu)$\times 10^{ - 4}$	OS (pu)$\times 10^{ - 4}$	US (pu)$\times 10^{ - 4}$
$M_{1}$	+ 25	–	− 0.6583	0.2029	− 1.424	0.83	− 0.0483	0.2484	− 1.132	0.3658	− 1.968	1.009	− 0.4569
	− 25	0.072	− 0.8273	0.2029	− 1.426	0.8255	− 0.0446	0.2374	− 1.268	0.3661	− 1.974	0.978	− 0.459
$M_{2}$	+ 25	–	− 0.7257	0.2341	− 1.311	0.8524	− 0.0539	0.2427	− 1.187	0.3745	− 1.852	1.017	− 0.4608
	− 25	–	− 0.727	0.1764	− 1.593	0.8187	− 0.0405	0.2429	− 1.194	0.3579	− 2.13	0.967	− 0.4552

It is found that the proposed controller managed to keep the frequency deviations in the two areas and the tie-line power deviation within an acceptable range when the system parameters change. It is noteworthy that the settling times of $\Delta F_{1}$, $\Delta P_{tie}$, and $\Delta P_{tie}$ didn’t change approximately with the system parameters change, so they weren’t included in the table. It is clear that the proposed controller has better transient response characteristics than MPA-Cascaded PIDA against parameters change.

## Conclusions

The study concludes that the proposed FOAPID/HS-PIDA cascaded controller provides substantial improvements in load frequency regulation for renewable–thermal hybrid systems. In 1% load disturbance tests, it achieved a settling time of 3.1 s versus 14.05 s, 17.92 s, and 11.9 s for TLBO PIDA, SCA PIDA, and Fuzzy PID, respectively, while reducing undershoot in ∆F_1_ by 80–83% and eliminating steady-state error in ∆F_2_ and tie-line power where competing methods failed. Under 20% load increase with renewable variability, competing controllers became unstable or exhibited steady-state errors, whereas the proposed method converged within 3.7 s and reduced overshoots by 81.6%, 88.8%, and 99.9% for ∆F_1_, ∆F_2_, and ∆P_tie_, respectively. SSE values dropped by more than 99% and RMSE values by up to 97%, confirming quantitative superiority. Compared to MPA-Cascaded PIDA, overshoots and undershoots were reduced by up to 89.85%, with settling times shortened to 6.2–6.3 s instead of 10–21 s. Robustness tests under ± 25% inertia variation verified stable performance with negligible deterioration in frequency response. As future work, the scope will be extended to include detailed stability studies in both frequency and time domains, as well as hardware-in-the-loop (HIL) validation to experimentally verify the simulation results and confirm the practical feasibility of the proposed cascaded control approach. Further, future works will also discuss the adaptability of utilizing different optimizers instead of HS algorithm to get more controllability in nonlinear systems.

## Data Availability

The datasets used and/or analyzed during the current study are available from the corresponding author on reasonable request.

## Abbreviations

LFC: Load frequency control
RES: Renewable energy sources
FOAPID: Fractional order adaptive proportional integral derivative
PIDA: Proportional integral derivative accelerated
HS: Harmony search
TLBO: Teaching learning based optimization
SCA: Sine cosine algorithm
ACE: Area control error
SSE: Sum of squares error
RMSE: Root mean square error
$$K_{p}$$: Proportional gain
$$K_{i}$$: Integral gain
$$K_{d}$$: Derivative gain
$K_{w1,} K_{w2, } K_{w3}$: Wind turbine gains
$T_{w1} , T_{w2}$: Wind turbine time constants
R: Speed regulation
B: Frequency bias factor
$T_{g}$: Governor time constant
$T_{t}$: Turbine time constant
$K_{l}$: Power system gain
$$T_{l}$$: Time constant
$$\Delta F$$: Frequency deviation
$\Delta P_{tie}$: Tie line power flow deviation
$$\Delta P_{L}$$: Load disturbance
$T_{12}$: Tie line synchronizing coefficient
$$K_{a}$$: Acceleration gain
$\lambda$: Fractional order of the integrator
$\mu$: Fractional order of the differentiator
$\gamma$: Learning rate of the adaptive mechanism
$T_{12}$: Tie line synchronizing coefficient
